# Machine Learning-Based Multiparametric Magnetic Resonance Imaging Radiomics Model for Preoperative Predicting the Deep Stromal Invasion in Patients with Early Cervical Cancer

**DOI:** 10.1007/s10278-023-00906-w

**Published:** 2024-01-10

**Authors:** Haowen Yan, Gaoting Huang, Zhihe Yang, Yirong Chen, Zhiming Xiang

**Affiliations:** 1 The First Affiliated Hospital of Jinan University, 510632 Guangzhou, China; 2 Department of Oncology, Guangzhou Panyu Central Hospital, 511400 Guangzhou, China; 3 Department of Gynecology and Oncology, Affiliated Cancer Hospital and Institute of Guangzhou Medical University, 510095 Guangzhou, China; 4 Department of Radiology, Guangzhou Panyu Central Hospital, 511400 Guangzhou, China; 5 Jinan University, 601# Huangpu Avenue West, Tianhe District, 510632 Guangzhou, China

**Keywords:** Cervical cancer, Deep stromal invasion, Radiomics, MRI

## Abstract

Deep stromal invasion is an important pathological factor associated with the treatments and prognosis of cervical cancer patients. Accurate determination of deep stromal invasion before radical hysterectomy (RH) is of great value for early clinical treatment decision-making and improving the prognosis of these patients. Machine learning is gradually applied in the construction of clinical models to improve the accuracy of clinical diagnosis or prediction, but whether machine learning can improve the preoperative diagnosis accuracy of deep stromal invasion in patients with cervical cancer was still unclear. This cross-sectional study was to construct three preoperative diagnostic models for deep stromal invasion in patients with early cervical cancer based on clinical, radiomics, and clinical combined radiomics data using the machine learning method. We enrolled 229 patients with early cervical cancer receiving RH combined with pelvic lymph node dissection (PLND). The least absolute shrinkage and selection operator (LASSO) and the fivefold cross-validation were applied to screen out radiomics features. Univariate and multivariate logistic regression analyses were applied to identify clinical predictors. All subjects were divided into the training set (*n* = 160) and testing set (*n* = 69) at a ratio of 7:3. Three light gradient boosting machine (LightGBM) models were constructed in the training set and verified in the testing set. The radiomics features were statistically different between deep stromal invasion < 1/3 group and deep stromal invasion ≥ 1/3 group. In the training set, the area under the curve (AUC) of the prediction model based on radiomics features was 0.951 (95% confidence interval (CI) 0.922–0.980), the AUC of the prediction model based on clinical predictors was 0.769 (95% CI 0.703–0.835), and the AUC of the prediction model based on radiomics features and clinical predictors was 0.969 (95% CI 0.947–0.990). The AUC of the prediction model based on radiomics features and clinical predictors was 0.914 (95% CI 0.848–0.980) in the testing set. The prediction model for deep stromal invasion in patients with early cervical cancer based on clinical and radiomics data exhibited good predictive performance with an AUC of 0.969, which might help the clinicians early identify patients with high risk of deep stromal invasion and provide timely interventions.

**Supplementary Information:**

The online version contains supplementary material available at 10.1007/s10278-023-00906-w.

## Introduction

Cervical cancer is the fourth most common malignancy and the fourth leading cause of cancer-associated death in females [1]. Previous evidence has reported that there will be 604,000 new cases of cervical cancer and 342,000 deaths in 2020, posing a serious threat to global women’s health all over the world [2]. Over the past few decades, increasing number of cervical cancer patients were detected at an early stage due to the spread of cervical cancer screening [3]. Deep stromal invasion is an important pathological factor associated with the treatments and prognosis of cervical cancer patients [4, 5]. Patients with moderate or 1/3 deep stromal invasion were recommended to receive adjuvant radiotherapy after radical hysterectomy (RH), especially for cervical cancer patients with vascular infiltration and other risk factors [5, 6]. At present, the diagnosis of deep stromal invasion is mainly confirmed by postoperative pathology data [7]. Accurate determination of deep stromal invasion before RH is of great value for early clinical treatment decision-making and improving the prognosis of these patients.

Magnetic resonance imaging (MRI) is a routine imaging examination method for diagnosis, staging, and monitoring of cervical cancer [8]. Currently, studies based on MRI features or quantitative imaging parameters were extracted by naked eyes, which can observe limited visual image gray scale, and some microscopic imaging features related to clinical results may be lost, hampering the accurate representation of tumor heterogeneity [9, 10]. The visual assessment of MRI features by trained radiologists is prone to interobserver variability and lacks generalizability across different institutions [11]. Radiomics is an emerging technology with quantitative features extracted from radiographic medical images by data-characterization algorithms, which is designed to develop prognostic prediction tools and treatment decision support tools in cancers [12]. The predictive value of radiomics using MRI data for preoperative lymph node metastasis, vascular invasion, and parastatal invasion of early cervical cancer has been confirmed previously [10, 13, 14]. Recently, Ren et al. constructed a MRI-based radiomics model to predict the preoperative deep stromal invasion, and the AUC of the model based on radiomics features constructed by logistics regression was 0.879, and combined with clinical features, the AUC was 0.886 [15]. Nonetheless, the predictive values of prediction models for preoperative deep stromal invasion in patients with early cervical cancer still need improving.

The conventional logistic regression model can only explore the linear associations, and nonlinear associations cannot be solved; the accuracy of the prediction models was not always good [16]. Lack of high-quality dataset algorithm training and development and proper validation using more updated methods might be major drawbacks in current clinical practices to predict preoperative deep stromal invasion in patients with early cervical cancer. In order to improve the accuracy of clinical diagnosis or prediction, machine learning is gradually applied in the construction of clinical models, which showed better effects than traditional models such as logistic regression [17, 18]. Machine learning involves the utilization of computer algorithms to derive predictive models from data, and these algorithms ascertain mathematical functions that elucidate the relationships between features within a given dataset [19]. Lately, increasing studies revealed that the integration of radiomics and machine learning enabled the development of classification models for targeted diagnosis of various diseases [19, 20]. However, there was no study combining radiomics and machine learning methods to construct prediction models for preoperative diagnosis of deep stromal invasion in patients with early cervical cancer. Light gradient boosting machine (GBM) is one of the machine learning methods that can reduce calculation time and allow missing values for prediction, which is more advantageous than the conventional logistic regression model [21]. Compared to deep learning and other traditional machine learning algorithms, LightGBM showed better generalization ability [22]. Whether LightGBM can improve the preoperative diagnosis accuracy of deep stromal invasion in patients with cervical cancer based on radiomics data was still unclear.

In the present study, the machine learning method was used to construct three preoperative diagnostic models for deep stromal invasion in patients with early cervical cancer based on clinical, radiomics, and clinical combined radiomics data, respectively. The predictive efficacy of different models was compared. The findings might help identify a novel tool for risk stratification of deep stromal invasion in patients with early cervical cancer in a quicker and more accurate manner. This might help guide the clinicians to make proper treatment adjustments for these patients and improve their prognosis.

## Methods

### Study Design and Population

This cross-sectional study enrolled 245 patients with early cervical cancer receiving RH combined with pelvic lymph node dissection (PLND) in the local hospital. The inclusion criteria were as follows: (1) patients’ age ≥ 18 years old, (2) patients with primary cervical cancer confirmed by pathology, (3) patients receiving RH combined with PLND, (4) patients who underwent MRI examination within 2 weeks before surgery, (5) patients with complete clinical data. The exclusion criteria were (1) patients with other malignant tumors, (2) patients undergoing palliative tumor resection, (3) pregnant or lactating women, (4) patients who received neoadjuvant therapy before surgery, and (5) MRI data does not meet the requirements of post-processing. After excluding participants who received neoadjuvant therapy before surgery, subjects receiving RH combined with PLND in other hospital, and patients with positive circumferential resection margin, 229 patients were included. This study was approved by the Ethics Committee of the local hospital. Informed consent was obtained from all individual participants included in the study.

### Radiomic Features Extraction

T_2_-weighted images and contrast-enhanced T1-weighted imaging were exported from the workstation of image storage and transmission system in Digital Imaging and Communications in Medicine format. A semi-automatic threshold classification method was used to select region of interest (ROI) of MRI using the 3D region growing GrowCut algorithm from the medical image analysis and visualization Slicer platform (3D-Slicer; version 4.3.1). Given a set of initial label points, the 3D-Slicer algorithm can automatically segment the remaining images through cellular automation, which achieves reliable and reasonably fast segmentation of moderately difficult objects in 2D and 3D using an iterative labeling procedure resembling competitive region growing [23]. Since the MRI were collected from different devices, the images were normalized before extraction, and all images were unified into a resolution of 1 × 1 mm by means of interpolation. ROI covered the entire tumor region. For each patient, a total of 2632 features (T2-weighted images + T1-weighted imaging) were extracted using the “PyRadiomics” package implemented in Python 3.11.1 (Supplementary Table 1). The features included first-order features (*n* = 18), texture features derived from texture matrices including grey-level co-occurrence matrix (*n* = 24), grey-level run length matrix (*n* = 16), grey-level size zone matrix (*n* = 16), grey-level dependence matrix (*n* = 14), neighboring gray tone difference matrix (*n* = 5) and shape-based (*n* = 14), wavelet transform features including first-order features (*n* = 144), grey-level co-occurrence matrix (*n* = 192), grey-level dependence matrix (*n* = 112), grey-level run length matrix (*n* = 128), grey-level size zone matrix (*n* = 128) and neighboring gray tone difference matrix (*n* = 40), and local binary pattern including first-order features (*n* = 90), grey-level co-occurrence matrix (*n* = 120), grey-level dependence matrix (*n* = 70), grey-level run length matrix (*n* = 80), and grey-level size zone matrix (*n* = 80) and neighboring gray tone difference matrix (*n* = 25).

### Clinical Variables

Age (years), body mass index (BMI, kg/m^2^), menopausal status (premenopausal, perimenopause or postmenopausal), the International Federation of Gynecology and Obstetrics (FIGO) staging (IA, IIA, IB, or IIB), marital status (married or unmarried), preterm birth history (yes or no), reproductive history (primipara or meningopara), history of abortion (yes or no), histological subtype (adenocarcinoma, squamous cell carcinoma or other), complicated with other diseases (yes or no), red blood cell (RBC), white blood cell (WBC), platelet (PLT), neutrophil percentage (NEU; %), lymphocyte percentage (LYM; %), monocyte percentage (MONO; %), eosinophil percentage (EOS; %), basophil percentage (BASO; %), NEU (10^9^/L), LYM (10^9^/L), MONO (10^9^/L), EOS (10^9^/L), BASO (10^9^/L), tumor size, carcinoembryonic antigen (CEA; normal or abnormal; ng/mL), squamous cell carcinoma antigen (SCC-Ag; normal or abnormal; ng/mL), carbohydrate antigen-125 (CA125; normal or abnormal; ng/mL), and carbohydrate antigen-199 (CA199; normal or abnormal; U/mL) were analyzed.

### Building Prediction Classifiers

**Fig. 1:**
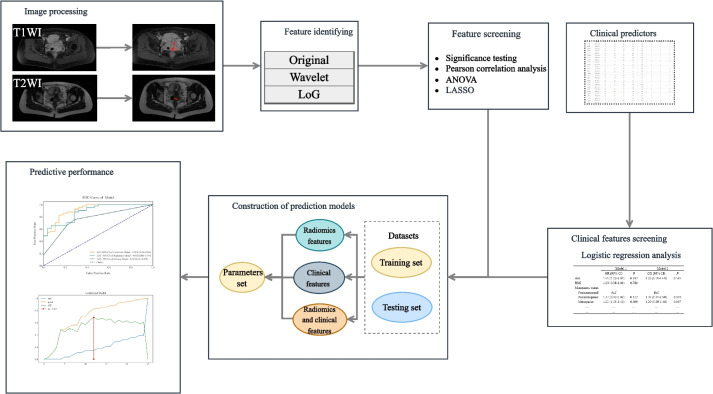
The proposed model’s whole architecture

The radiomics features were extracted after image segmentation on the original MRI image to delineate the ROI, and features with statistical significance (*P* < 0.05) were included (SciPy tool in Python version 1.10.0). Then Pearson’s correlation coefficient was applied; when the Pearson correlation coefficient between the two features > 0.85, the features with higher *P*-value were excluded (Pandas tool in Python version 1.5.3). Further, the analysis of variance (ANOVA) was applied to select the top 15 radiomics features with high variance (scikit-learn tool in Python version 1.2.1). Next, the least absolute shrinkage and selection operator (LASSO) and the fivefold cross-validation were applied to further screen out features (coefficent ≠ 0). Univariable and multivariate logistic regression analyses were applied to identify clinical predictors associated with the deep stromal invasion in patients with early cervical cancer, and variables with statistical association with deep stromal invasion in patients with early cervical cancer were included as clinical predictors (*P* < 0.05). All subjects were randomly divided into the training set (*n* = 160) and testing set (*n* = 69) at a ratio of 7:3. Three LightGBM models were constructed in the training set: a radiomics model constructed with radiomics features alone (model 1), a clinical model constructed with clinic features alone (model 2), and a combined model constructed with the combination of radiomics features and clinical predictor (model 3). The parameters set for training each model are shown in Table 1. During the training of each model, optuna ultra parameter optimization tool was adopted to optimize the parameters, the optimized model was used to verify in the training set, and the corresponding evaluation indexes were calculated. The predictive performances of the models were verified in the testing set. The proposed model’s whole architecture is exhibited in Fig. 1. The pseudocode for the proposed work was shown as follows: 
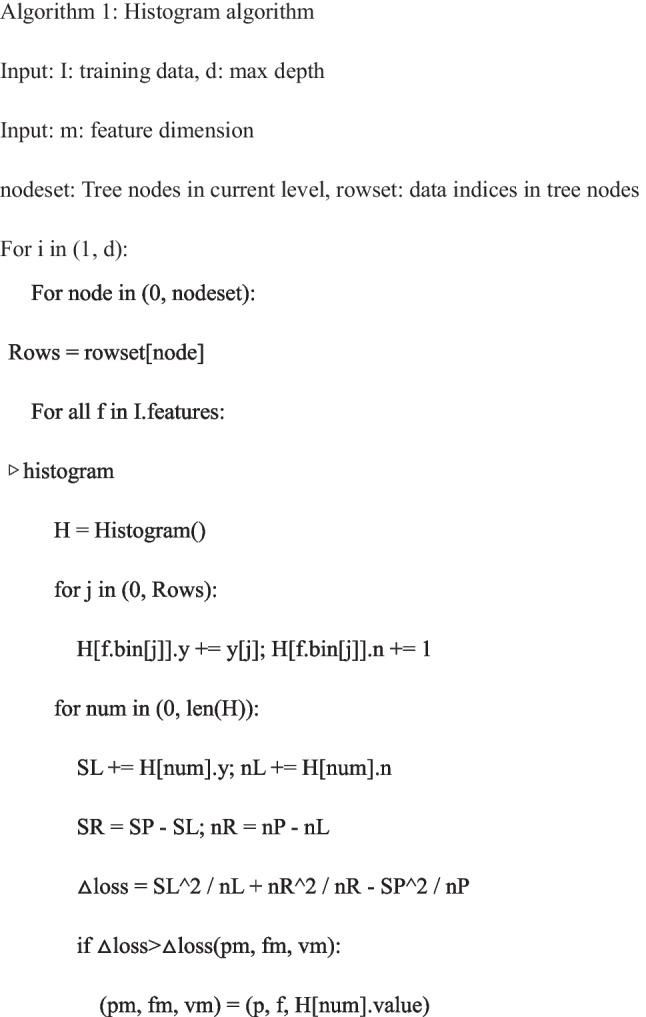

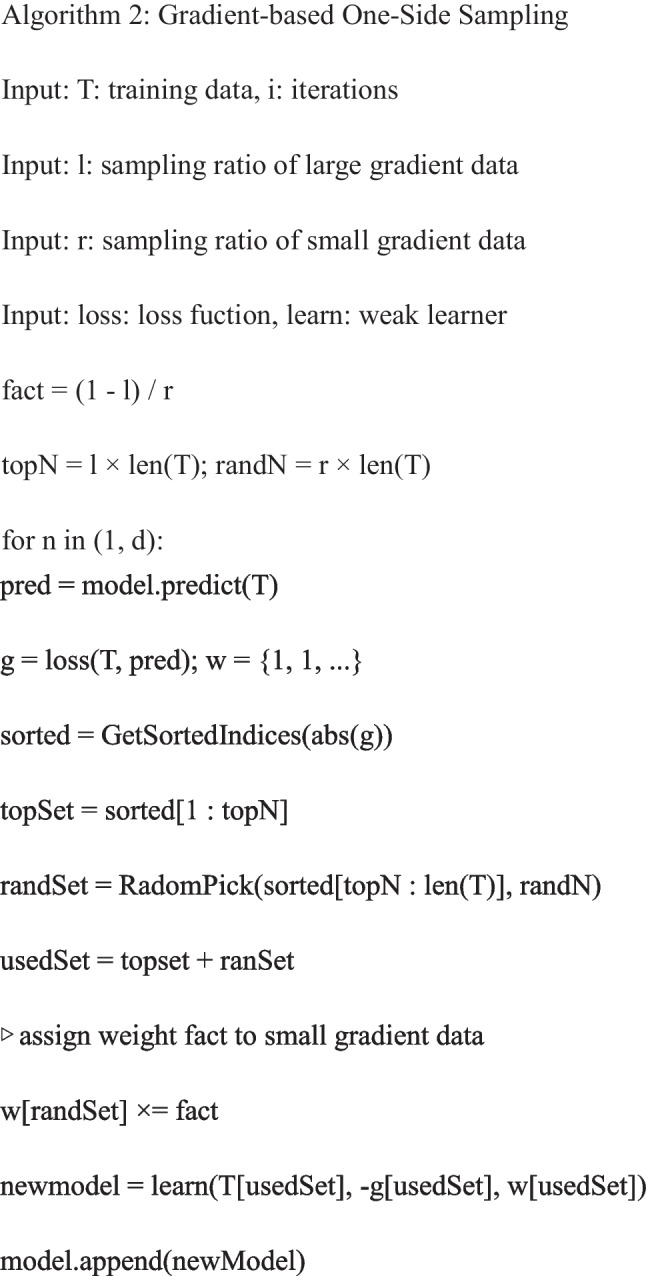

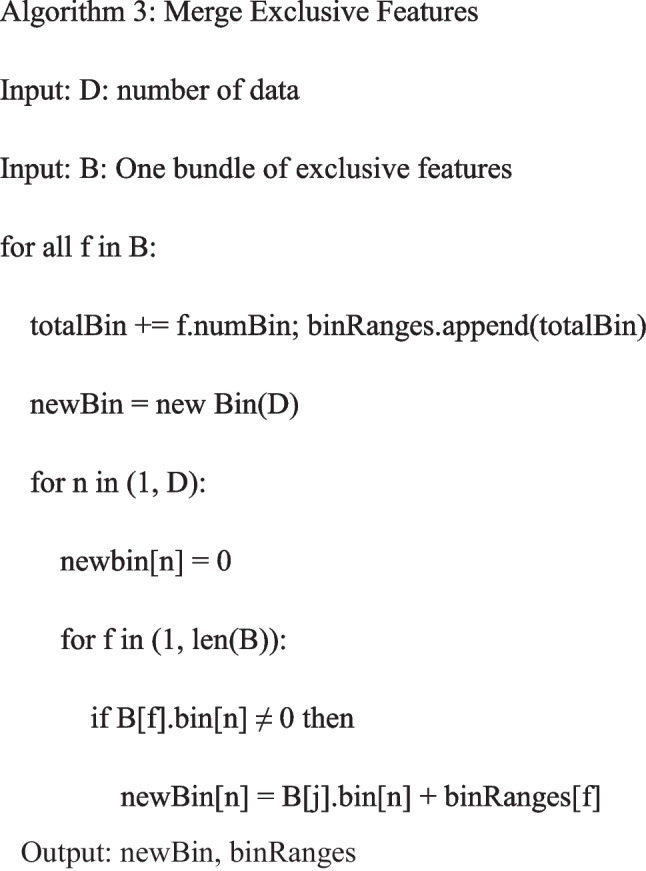

**Table 1:** The parameters used for training each prediction model

Parameters	Radiomics features	Clinical features	Radiomics and clinical features
boosting_type	gbdt	gbdt	gbdt
objective	binary	binary	binary
metric	auc	auc	auc
max_depth	6	8	5
num_leaves	12	14	13
min_data_in_leaf	15	7	7
max_bin	127	31	31
feature_fraction	0.9	0.7	0.7
bagging_fraction	0.6	0.9	0.8
lambda_l1	0.006587301	0.057721404	0.002437294
lambda_l2	0.029924765	0.000530493	0.000600306
learning_rate	0.01	0.01	0.01
random_state	3000	3000	3000

### Measurement of the Performance of the Prediction Model

The proposed model more accurately predicted the deep stromal invasion in patients with early cervical cancer. The robustness of the model was assessed in the training set and the testing set. F1 score, accuracy, sensitivity, specificity, negative predictive value (NPV), positive predictive value (PPV), and area under the curve (AUC) were employed to evaluate the predictive values of the models. The receiver operator characteristic (ROC) curves and Kolmogorov–Smirnov (KS) curves were plotted.

The accuracy assessment parameter is calculated:

$$\mathrm{F}1\mathrm{ score}=\frac{2\times \mathrm{SN}\times \mathrm{PRE}}{\mathrm{SN}+\mathrm{PRE}}$$

$$\mathrm{Accurancy}=\frac{\mathrm{TP}+\mathrm{TN}}{\mathrm{TP}+\mathrm{TN}+\mathrm{FP}+\mathrm{FN}}$$

$$\mathrm{Sensitivity}={^{\mathrm{TP}}/ _{(\mathrm{TP}+\mathrm{FN})}}$$

$$\mathrm{Specificity}= {^{\mathrm{TN}}/_{(\mathrm{TN}+\mathrm{FP})}}$$

SN, sensitivity; TP, true positive; TN, true negative; FP, false positive; FN, false negative; PRE, TP/(TP + FP).

### Statistical Analysis

The measurement data of normal distribution were expressed as mean and standard deviation (Mean (SD)), and *t* test was used to compare the differences between the two groups. Median and quartiles were used to describe the distribution of non-normally-distributed measurement data, and Wilcoxon rank sum test was used to compare the difference between the two groups. The enumeration data were displayed using the number of cases and percentages, and the chi-square test was used to compare differences between groups. The radiomics features were extracted, and features were selected via Pearson’s correlation coefficient, ANOVA, LASSO regression analysis, and the fivefold cross-validation. Univariable and multivariate logistic regression analyses were applied to identify clinical predictors associated with the deep stromal invasion in patients with early cervical cancer. All subjects were randomly split into the training set (*n* = 160) and testing set (*n* = 69) at a ratio of 7:3. Three LightGBM models were constructed in the training set: model 1 included radiomics features, model 2 included clinical predictors, and model 3 included radiomics features and clinical predictors. The models were verified in the testing set. The ROC and KS curves were plotted. The confidence level was alpha = 0.05. R (Institute for Statistics and Mathematics, Vienna, Austria) was used for data analysis. Python 3.11.1 was used for radiomics features extraction and model construction.

## Results

### Identification of Predictors in the Models for Deep Stromal Invasion in Patients with Early Cervical Cancer

**Fig. 2:**
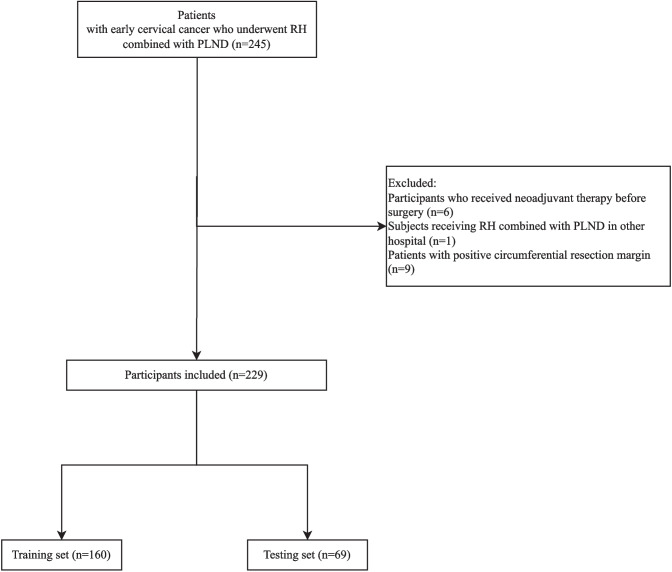
The screen process of the participants

In total, 245 patients with early cervical cancer who underwent RH combined with PLND in the local hospital were enrolled. Among them, participants who received neoadjuvant therapy before surgery (*n* = 6), subjects receiving RH combined with PLND in other hospital (*n* = 1), and patients with positive circumferential resection margin (*n* = 9) were excluded. Finally, 229 patients were included. The screen process of participants is shown in Fig. 2.

**Fig. 3:**
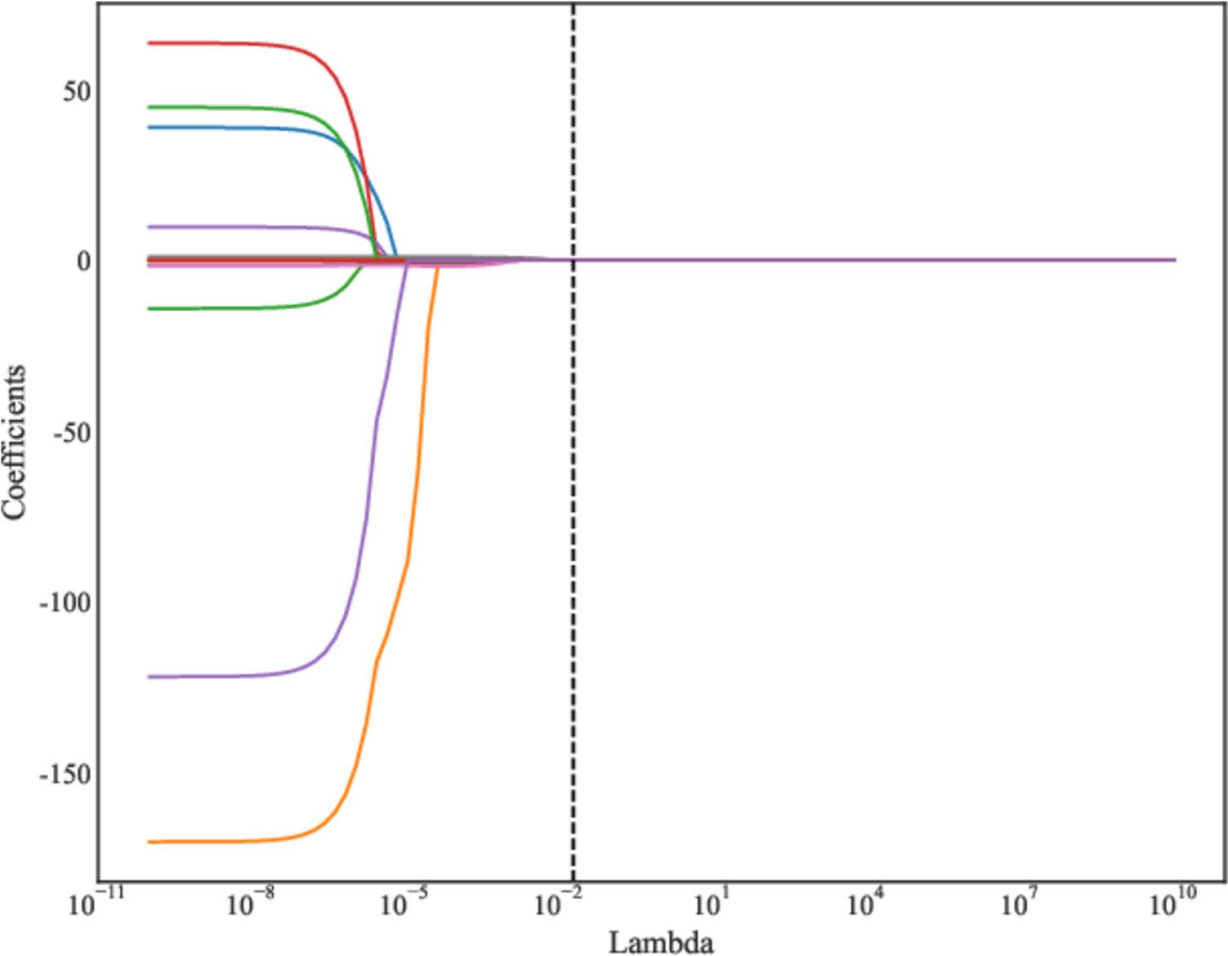
The results of LASSO regression analysis for radiomics features

**Fig. 4:**
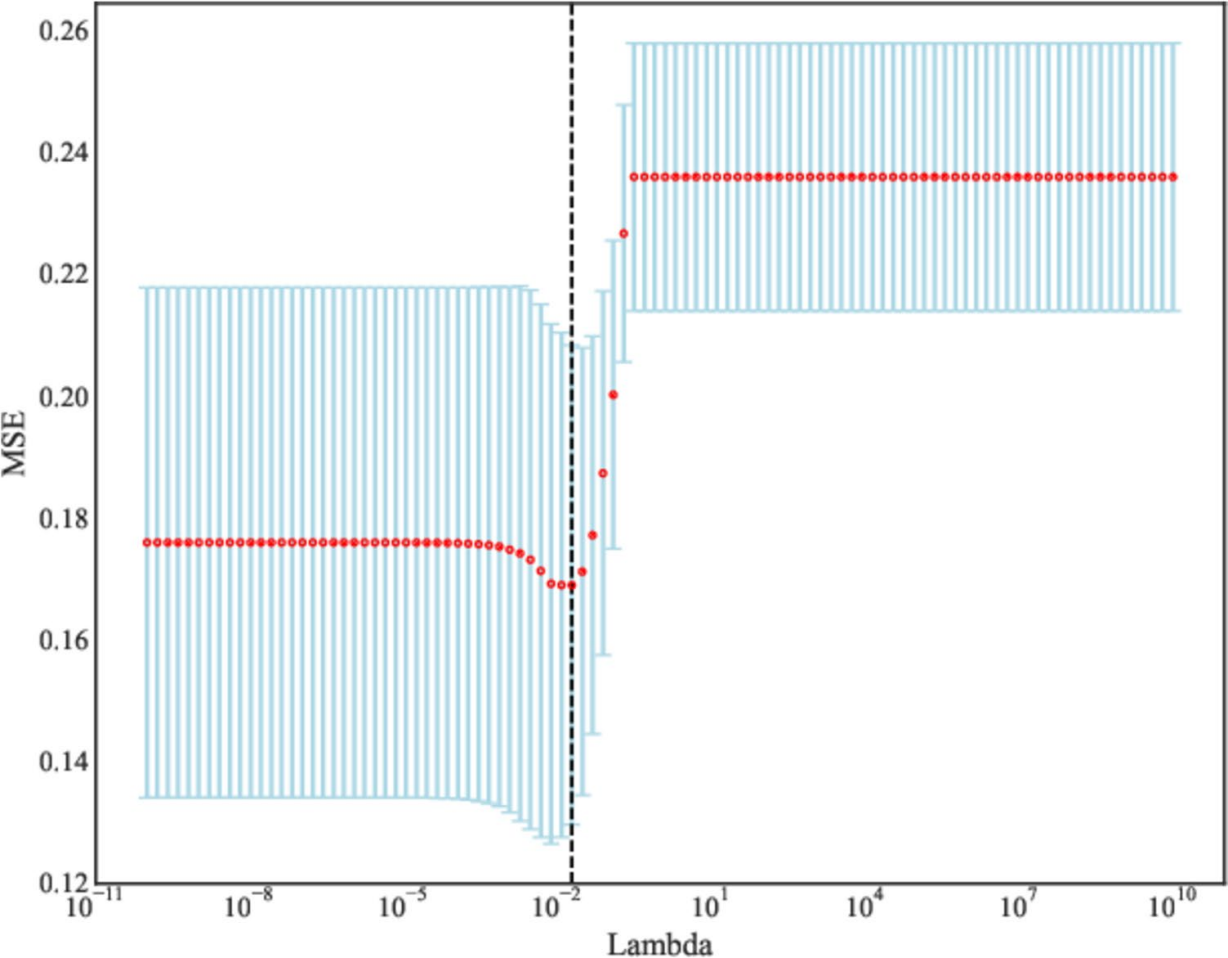
The optimal Lambda value of LASSO regression analysis

**Fig. 5:**
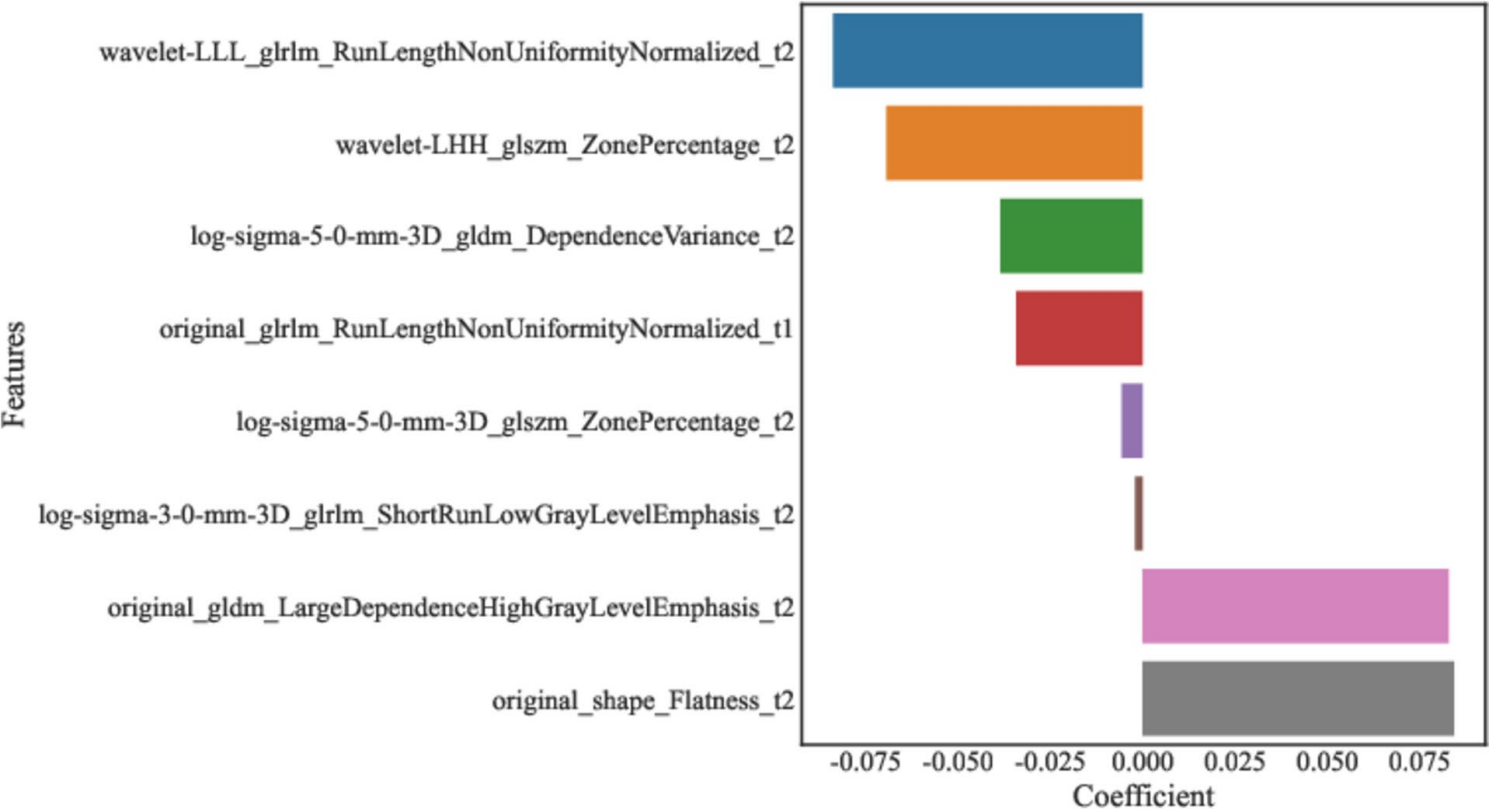
The coefficients of features screened out by LASSO regression analysis

A total of 2632 features were extracted from MRI, and those with statistical significance (*P* < 0.05) were kept. When the Pearson correlation coefficient between the two features were > 0.85, the features with higher *P*-value were excluded. Further, the ANOVA was applied to select the top 15 radiomics features with high variance. Finally, LASSO regression analysis was applied to screen out the features (Fig. 3, Table 2). We used fivefold cross-validation to find the optimal value of regularization parameter lambda with mean square error, and MSE was changed with lambda. The optimal lambda value was used for variable selection and was 0.019179102616724848 (Fig. 4). The coefficients of features finally included are exhibited in Table 2 and Fig. 5.

**Table 2:** The radiomics features associated with deep stromal invasion in patients with early cervical cancer screened by LASSO

Features	Coefficient
wavelet-LLL_glrlm_RunLengthNonUniformityNormalized_t2	−0.08381
wavelet-LHH_glszm_ZonePercentage_t2	−0.06938
log-sigma-5–0-mm-3D_gldm_DependenceVariance_t2	−0.03851
original_glrlm_RunLengthNonUniformityNormalized_t1	−0.03422
log-sigma-5–0-mm-3D_glszm_ZonePercentage_t2	−0.00558
log-sigma-3–0-mm-3D_glrlm_ShortRunLowGrayLevelEmphasis_t2	−0.00195
original_gldm_LargeDependenceHighGrayLevelEmphasis_t2	0.082895
original_shape_Flatness_t2	0.084346

*LASSO* least absolute shrinkage and selection operator

As presented in Table 3, age, postmenopausal, FIGO-IIA, LYM, tumor size, SCC-Ag, and CA125 might be associated with deep stromal invasion in patients with early cervical cancer. Multivariate logistical regression analysis revealed that FIGO-IIA (OR = 2.43, 95% CI 1.36–4.37), FIGO-IB (OR = 1.87, 95% CI 1.05–3.33) and FIGO-IIB (OR = 3.42, 95% CI 1.28–9.15), and SCC-Ag (OR = 1.38, 95% CI 1.19–1.59) were correlated with deep stromal invasion in patients with early cervical cancer.

**Table 3:** Clinical predictors for deep stromal invasion in patients with early cervical cancer

	Univariate		Multivariable	
Characteristics	Odd ratio	*P*	Odd ratio	*P*
Age (years)	1.01 (1.00–1.02)	0.012	1.00 (0.99–1.01)	0.585
BMI	1.00 (0.98–1.03)	0.780		
Menopausal status				
Premenopausal	Ref		Ref	
Perimenopause	1.37 (0.92–2.03)	0.122	1.37 (0.95–1.96)	0.091
Postmenopausal	1.22 (1.05–1.42)	0.009	1.20 (0.99–1.46)	0.067
FIGO staging				
IA				
IIA	2.46 (1.26–4.79)	0.009	2.43 (1.36–4.37)	0.003
IB	1.78 (0.93–3.40)	0.085	1.87 (1.05–3.33)	0.035
IIB	2.72 (0.89–8.30)	0.081	3.42 (1.28–9.15)	0.016
Marital status				
Married	Ref			
Unmarried	1.45 (0.56–3.76)	0.446		
Preterm birth history				
No	Ref			
Yes	0.53 (0.21–1.37)	0.192		
Reproductive history				
Primipara	Ref			
Meningopara	0.89 (0.55–1.43)	0.621		
History of abortion				
No	Ref			
Yes	0.93 (0.80–1.09)	0.379		
Histological subtype				
Adenocarcinoma	Ref			
Squamous cell carcinoma	1.10 (0.90–1.34)	0.356		
Other	1.16 (0.82–1.64)	0.406		
Complicated with other diseases				
No	Ref			
Yes	0.96 (0.81–1.15)	0.677		
RBC	0.91 (0.80–1.04)	0.165		
WBC	1.02 (0.99–1.05)	0.208		
PLT	1.00 (1.00–1.00)	0.193		
NEU%	1.01 (1.00–1.01)	0.111		
LYM%	0.99 (0.98–1.00)	0.046	0.99 (0.99–1.00)	0.060
MONO%	0.98 (0.94–1.03)	0.471		
EOS%	1.02 (0.98–1.06)	0.343		
BASO%	1.14 (0.85–1.52)	0.378		
NEU	1.03 (0.99–1.06)	0.140		
LYM	0.97 (0.86–1.09)	0.586		
MONO	0.91 (0.76–1.09)	0.320		
EOS	1.23 (0.76–2.00)	0.407		
BASO	1.54 (0.37–6.43)	0.556		
Tumor size	1.01 (1.01–1.02)	< 0.001	1.00 (1.00–1.01)	0.165
CEA				
Normal	Ref			
Abnormal	1.18 (0.93–1.49)	0.174		
SCC-Ag				
Normal	Ref			
Abnormal	1.52 (1.32–1.75)	< 0.001	1.38 (1.19–1.59)	< 0.001
CA125				
Normal	Ref		Ref	
Abnormal	1.31 (1.02–1.68)	0.033	1.21 (0.96–1.53)	0.105
CA199				
Normal	Ref			
Abnormal	1.15 (0.91–1.44)	0.242		

*BMI* body mass index, *FIGO* International Federation of Gynecology and Obstetrics, *RBC* red blood cell, *WBC* white blood cell, *PLT* platelet, *NEU* neutrophil, *LYM* lymphocyte, *MONO* monocyte, *EOS* eosinophil, *BASO* basophil, *CEA* carcinoembryonic antigen, *SCC-Ag* squamous cell carcinoma antigen, *CA125* carbohydrate antigen-125, *CA199* carbohydrate antigen-199

### Construction of the Prediction Models for Deep Stromal Invasion in Patients with Early Cervical Cancer

All the samples were randomly divided into the training set (*n* = 160) and the testing set (*n* = 69). There was no statistical difference between the data in the training set and testing set according to the results of equilibrium test (all* P* > 0.05) (Table 4). The numbers of samples with deep stromal invasion < 1/3 and deep stromal invasion ≥ 1/3 in different dataset are presented in Table 5. The percentages of patients with different FIGO staging and abnormal SCC-Ag (11.86% vs 54.46%) were statistically different between deep stromal invasion < 1/3 group and deep stromal invasion ≥ 1/3 group. The radiomics features were also statistically different between deep stromal invasion < 1/3 group and deep stromal invasion ≥ 1/3 group (Table 6).

**Table 4:** Comparisons of the variables in the training set and the testing set

Variables	Training set (*n* = 160)	Testing set (*n* = 69)	*P*
FIGO staging			0.930
IA	2 (1.25)	1 (1.45)	
IIA	30 (18.75)	13 (18.84)	
IB	127 (79.38)	55 (79.71)	
IIB	1 (0.62)	0 (0.00)	
SCC-Ag (abnormal)	62 (38.75)	33 (47.83)	0.257
wavelet.LHH_glszm_ZonePercentage_t2 (median [IQR])	0.00 [0.00, 0.00]	0.00 [0.00, 0.00]	0.495
wavelet.LLL_glrlm_RunLengthNonUniformityNormalized_t2 (median [IQR])	0.11 [0.08, 0.14]	0.11 [0.08, 0.15]	0.905
original_glrlm_RunLengthNonUniformityNormalized_t1 (median [IQR])	0.09 [0.07, 0.12]	0.09 [0.07, 0.12]	0.739
original_shape_Flatness_t2 (mean (SD))	0.56 (0.13)	0.56 (0.15)	0.911
original_gldm_LargeDependenceHighGrayLevelEmphasis_t2 (median [IQR])	2117.57 [1826.07, 2412.38]	2186.58 [1891.59, 2399.83]	0.443
log.sigma.3.0.mm.3D_glrlm_ShortRunLowGrayLevelEmphasis_t2 (median [IQR])	0.11 [0.10, 0.13]	0.11 [0.10, 0.13]	0.609
log.sigma.5.0.mm.3D_gldm_DependenceVariance_t2 (median [IQR])	27.99 [25.43, 30.79]	28.30 [25.92, 30.64]	0.808
log.sigma.5.0.mm.3D_glszm_ZonePercentage_t2 (median [IQR])	0.00 [0.00, 0.00]	0.00 [0.00, 0.00]	0.573
Deep stromal invasion ≥ 1/3	101 (63.12)	41 (59.42)	0.598

*SD* standard deviation, *FIGO* International Federation of Gynecology and Obstetrics, *SCC-Ag* squamous cell carcinoma antigen

**Table 5:** The numbers of samples with deep stromal invasion < 1/3 and deep stromal invasion ≥ 1/3 in different dataset

Datasets	Sample size	Deep stromal invasion < 1/3	Deep stromal invasion ≥ 1/3
Total	229	87	142
Training set	160	59	101
Testing set	69	28	41

**Table 6:** Comparisons of variables of patients between deep stromal invasion < 1/3 group and deep stromal invasion ≥ 1/3 group

Variables	Deep stromal invasion < 1/3 (*n* = 59)	Deep stromal invasion ≥ 1/3 (*n* = 101)	*P*
FIGO staging			0.002
IA	2 (3.39)	0 (0.00)	
IIA	3 (5.08)	27 (26.73)	
IB	54 (91.53)	73 (72.28)	
IIB	0 (0.00)	1 (0.99)	
SCC-Ag (abnormal) (%)	7 (11.86)	55 (54.46)	< 0.001
wavelet.LHH_glszm_ZonePercentage_t2 (median [IQR])	0.00 [0.00, 0.00]	0.00 [0.00, 0.00]	< 0.001
wavelet.LLL_glrlm_RunLengthNonUniformityNormalized_t2 (median [IQR])	0.14 [0.13, 0.17]	0.10 [0.07, 0.12]	< 0.001
original_glrlm_RunLengthNonUniformityNormalized_t1 (median [IQR])	0.11 [0.10, 0.14]	0.08 [0.07, 0.10]	< 0.001
original_shape_Flatness_t2 (mean (SD))	0.49 (0.14)	0.60 (0.11)	< 0.001
original_gldm_LargeDependenceHighGrayLevelEmphasis_t2 (median [IQR])	1827.87 [1358.60, 2036.52]	2300.27 [2067.12, 2464.69]	< 0.001
log.sigma.3.0.mm.3D_glrlm_ShortRunLowGrayLevelEmphasis_t2 (median [IQR])	0.13 [0.11, 0.15]	0.11 [0.09, 0.13]	< 0.001
log.sigma.5.0.mm.3D_gldm_DependenceVariance_t2 (median [IQR])	30.71 [28.31, 34.02]	26.73 [24.52, 29.08]	< 0.001
log.sigma.5.0.mm.3D_glszm_ZonePercentage_t2 (median [IQR])	0.00 [0.00, 0.00]	0.00 [0.00, 0.00]	< 0.001

*FIGO* International Federation of Gynecology and Obstetrics, *SCC-Ag* squamous cell carcinoma antigen

### Evaluation of the Predictive Performance of the Prediction Models for Deep Stromal Invasion in Patients with Early Cervical Cancer

**Fig. 6:**
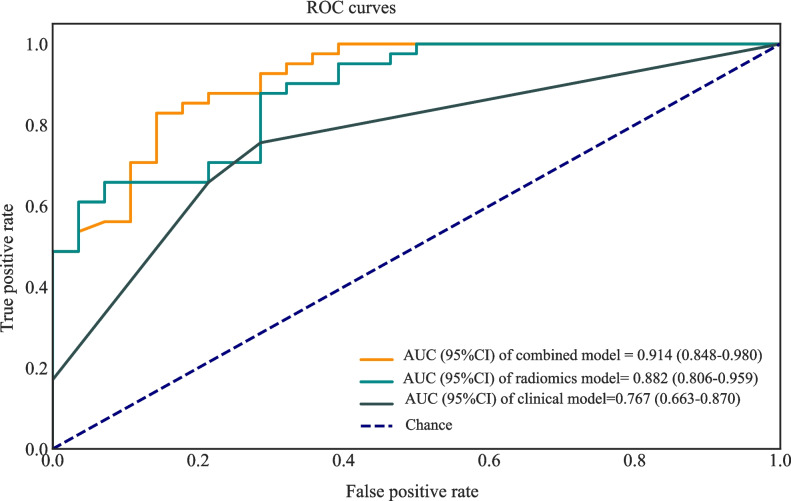
The ROC curves showing the AUCs of different models in the testing set

**Fig. 7:**
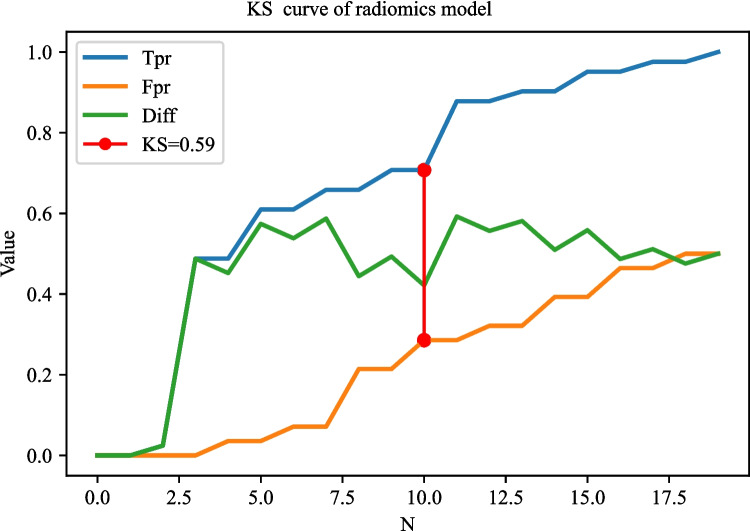
The KS curves of the prediction model based on radiomics features

**Fig. 8:**
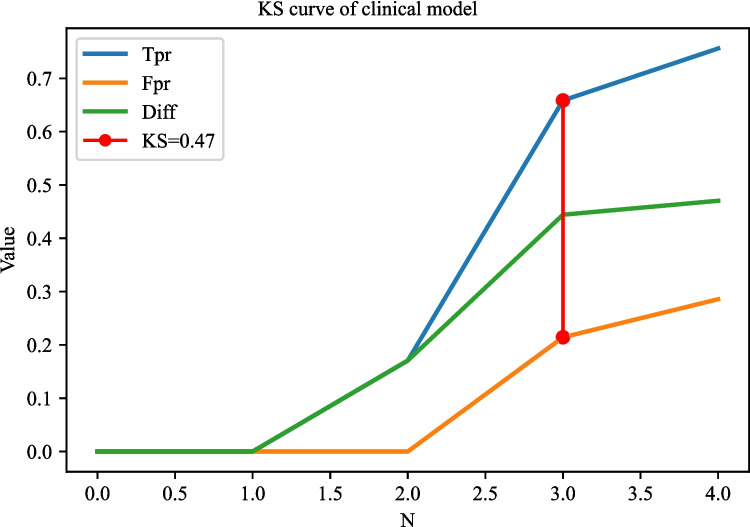
The KS curves of the prediction model based on clinical predictors

**Fig. 9:**
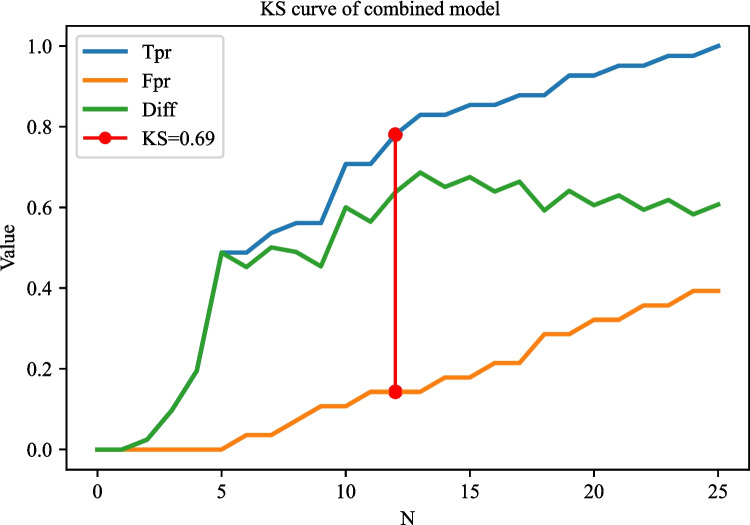
The KS curves of the prediction model based on radiomics features combined with clinical predictors

**Fig. 10:**
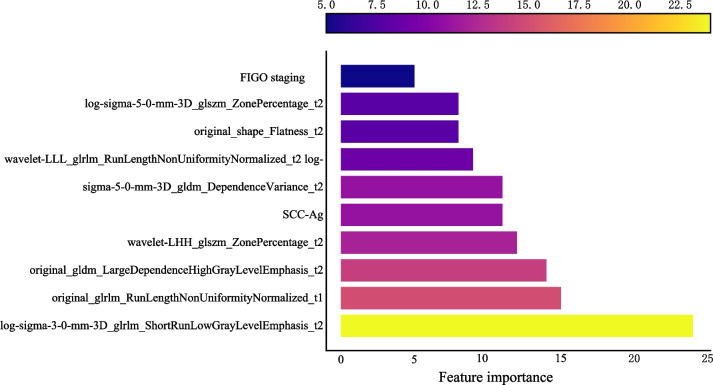
The variable importance of all the predictors in the prediction model based on radiomics features combined with clinical predictors

The AUC of the prediction model based on radiomics features was 0.951 (95% CI 0.922–0.980) in the training set. The AUC of the prediction model based on clinical predictors was 0.769 (95% CI 0.703–0.835) in the training set. The AUC of the prediction model based on radiomics features and clinical predictors was 0.969 (95% CI 0.947–0.990) in the training set (Table 7). The AUC of the prediction model based on radiomics features and clinical predictors was 0.914 (95% CI 0.848–0.980) in the testing set (Table 7, Fig. 6). The KS curves of the prediction models based on radiomics features, clinical predictors, and radiomics features combined with clinical predictors were plotted. The KS test was used to assess the agreement between the predicted and actual probabilities of deep stromal invasion and higher KS values indicating greater ability of the model to discriminate the samples. Generally, KS > 0.2 denotes a strong risk differentiation ability of the model developed. The KS values of the prediction models based on radiomics features, clinical predictors, and radiomics features combined with clinical predictors were 0.59 (Fig. 7), 0.47 (Fig. 8), and 0.69 (Fig. 9), respectively. The variable importance of all the predictors in the prediction model based on radiomics features combined with clinical predictors is presented in Fig. 10.

**Table 7:** The predictive values of the models

Models	Cut-off	Sensitivity	Specificity	PPV	NPV	F1 score	Accuracy	AUC (95% CI)
Training set								
Radiomics features	0.546	0.871	0.881	0.926	0.800	0.892	0.875	0.951 (0.922–0.980)
Clinical predictors	0.630	0.683	0.847	0.885	0.610	0.771	0.744	0.769 (0.703–0.835)
Radiomics and clinical predictors	0.623	0.901	0.932	0.958	0.846	0.929	0.912	0.969 (0.947–0.990)
Testing set								
Radiomics features	0.571	0.878	0.714	0.818	0.800	0.833	0.812	0.882 (0.806–0.959)
Clinical predictors	0.630	0.756	0.714	0.795	0.667	0.775	0.739	0.767 (0.663–0.870)
Radiomics and clinical predictors	0.633	0.829	0.857	0.895	0.774	0.861	0.841	0.914 (0.848–0.980)

*NPV* negative predictive value, *PPV* positive predictive value, *AUC* area under the curve

## Discussion

The present study constructed three preoperative diagnostic models for deep stromal invasion in patients with early cervical cancer based on clinical, radiomics, and clinical combined radiomics data based on machine learning method. The model combined with radiomics features and clinical predictors showed better predictive performance than the prediction models based on radiomics features or clinical predictors. The findings might provide an effective tool to help clinicians early identify patients with the deep stromal invasion and guide the treatments accordingly.

Previously, there were several prediction models based on MRI data for deep stromal invasion in patients with cervical cancer. Song et al. constructed a prediction model based on amide proton transfer weighted imaging combined with dynamic contrast-enhanced MRI and found that Ktrans + SCC-Ag had the AUC of 0.819 for predicting deep stromal invasion in patients with IB1-IIA1 cervical cancer [24]. Another prospective multicenter study constructed a preoperative prediction model for deep stromal invasion in women with invasive cervical cancer using 2D and 3D ultrasound and showed an AUC of 0.93 [25]. These models mostly constructed based on the conventional logistic regression model, which can only explore the linear associations, and the predictive ability still needs improvement [16]. To use the machine learning algorithm to train and validate the prediction model might help improve the predictive accuracy of deep stromal invasion in patients with early cervical cancer. In our study, the model based on radiomics features had an AUC of 0.951, and the AUC of the model based on radiomics features and clinical predictors was 0.969. The models presented better predictive performance for deep stromal invasion in patients with early cervical cancer than previous models. The detailed information on database, computational complexity, and reliability of our model and previous prediction models are exhibited in Table 8. MRI had the advantages of relatively low cost, high spatial resolution and contrast of pelvic tissues and organs, and no radiation [26, 27]. MRI was highly individual specific and non-invasive, which has been applied to clinical decision support for the improvement of the screening accuracy, diagnosis, and prognosis prediction [28]. The prediction model in our study was constructed using LightGBM, which used histogram-based segmentation algorithm instead of presort traversal algorithm to reduce the number of features by gradient-based one-side sampling (GOSS) and exclusive feature bundling (EFB) [29]. LightGBM had higher efficiency and accuracy [30] and better generalization ability [22]. The model combining LightGBM methods and MRI in the current study might provide a convenient and easy tool for early identification of those at a high risk of deep stromal invasion in patients with early cervical cancer. The accuracy for predicting deep stromal invasion in patients with early cervical cancer was improved compared to previous models, which might help guide the treatments options of these patients with high risk of deep stromal invasion, and early interventions might improve their prognosis.

**Table 8:** Comparisons of our prediction model and previous prediction models for deep stromal invasion in patients with early cervical cancer

Models	Samples (*n*)	Database	Space complexity	AUC	Sensitivity	Specificity
Our model (LightGBM)	229	Guangzhou Panyu Central Hospital	Memory cost O (nfeature × ndata)	0.969	0.901	0.932
			Calculation of split gain O (nbin × nfeature)			
			Exclusive feature bundling O (ndata × nfeature) → O (ndata × nbundle)			
Pálsdóttir et al. (logistic regression)	104	Lund University Hospital and Karolinska University Hospital	O (nfeature)	0.930	0.905	0.972
Ren et al. (logistic regression)	234	Peking Union Medical College Hospital	O (nfeature)	0.886	0.879	0.846

*AUC* area under the curve, *LightGBM* light gradient boosting machine

MRI is a vital exam for the initial assessment of loco-regional involvement of cervical cancer. In previous studies, multiple studies found that MRI was applied to evaluate the early response to radiochemotherapy before image-guided brachytherapy in patients with locally advanced cervical cancer [31]. Multiparametric MRI–derived radiomics was also applied for the prediction of disease-free survival in early-stage squamous cervical cancer [32]. Multimodal MRI was reported to have good diagnostic value for the discrimination of metastatic and non-metastatic pelvic lymph nodes in cervical cancer [33]. Another prospective preliminary study applied the synthetic MRI to evaluate the prognostic factors in cervical cancer [34]. These studies gave support to the results of this study, which elucidated that MRI-derived radiomics features were important predictors for deep stromal invasion in patients with early cervical cancer. Cancer staging is an essential index for the diagnosis, prognosis, and treatment of cervical cancer [35]. The FIGO staging system was widely applied in cervical cancer [36], which was reported to be associated with the treatment outcomes in early-stage cervical cancer patients [37]. Herein, the FIGO staging system was also found to be an important predictor for deep stromal invasion in patients with early cervical cancer. Another predictor for deep stromal invasion in patients with early cervical cancer in this study was SCC-Ag. This was allied by previous evidence. SCC-Ag was used in outcome prediction after concurrent chemo-radiotherapy and treatment decisions for patients with cervical cancer [38]. SCC-Ag changes in patients with locally advanced cervical cancer were one of the parameters of prognostic evaluation [39].

The current study compared the predictive abilities of three preoperative diagnostic models using the machine learning method for preoperative non-invasive diagnosis of deep stromal invasion in patients with early cervical cancer based on clinical, radiomics, and clinical combined radiomics data, respectively. The predicting performance of the model for deep stromal invasion in patients with early cervical cancer based on clinical combined radiomics data was good. The findings might provide a tool to help clinicians identify deep stromal invasion in patients with early cervical cancer and formulate treatment strategies accordingly. There were several limitations in this study. Firstly, the participants were from a single center, and there might be selection bias. Secondly, the MRI images were collected from different devices, which might have a potential impact on the stability of radiomics features. Therefore, the images were normalized before feature extraction, and all images were unified to a resolution of 1 × 1 mm. The standardization process was considered a useful way to promote good feature robustness in cervical cancer. In recent years, more and more deep learning methods such as automated in-depth feature learning algorithm [40] and a deep convolutional neural network-based approach [41] were widely applied for disease prediction and prognosis evaluation. These methods are unsupervised active learning, which increase efficiency and accuracy of diseases and prognosis prediction including cancers [42]. The future of applied deep learning in cervical cancer might help integrate medical images and clinical data to construct more reliable prediction models. In the future, more well-designed studies using deep learning methods were needed to verify the results in this study.

## Conclusions

The AUC values of the prediction model for deep stromal invasion in patients with early cervical cancer based on clinical and radiomics data were 0.969 in the training set and 0.914 in the testing set, which exhibited good predictive performance than previous prediction models. The prediction model might help the clinicians early and accurately identify patients with high risk of deep stromal invasion and provide timely interventions.

### Supplementary Information

Below is the link to the electronic supplementary material.

Supplementary file1 (DOCX 41 KB)

## Data Availability

Data will be made available on request.
