# Supplementary material for: Machine Learning-Based Multiparametric Magnetic Resonance Imaging Radiomics Model for Preoperative Predicting the Deep Stromal Invasion in Patients with Early Cervical Cancer
Source: J Imaging Inform Med. 2024 Jan 10;37(1):230–46. doi: 10.1007/s10278-023-00906-w (PMC11266323; doi:10.1007/s10278-023-00906-w)
Supplement: Supplementary file 1 — Supplementary file1 (DOCX 41 KB) [file 10278_2023_906_MOESM1_ESM.docx]

Supplementary Table 1 The detailed information of observed radiomics features

| 1 | log-sigma-1-0-mm-3D_firstorder_10Percentile |
| --- | --- |
| 2 | log-sigma-1-0-mm-3D_firstorder_90Percentile |
| 3 | log-sigma-1-0-mm-3D_firstorder_Energy |
| 4 | log-sigma-1-0-mm-3D_firstorder_Entropy |
| 5 | log-sigma-1-0-mm-3D_firstorder_InterquartileRange |
| 6 | log-sigma-1-0-mm-3D_firstorder_Kurtosis |
| 7 | log-sigma-1-0-mm-3D_firstorder_Maximum |
| 8 | log-sigma-1-0-mm-3D_firstorder_Mean |
| 9 | log-sigma-1-0-mm-3D_firstorder_MeanAbsoluteDeviation |
| 10 | log-sigma-1-0-mm-3D_firstorder_Median |
| 11 | log-sigma-1-0-mm-3D_firstorder_Minimum |
| 12 | log-sigma-1-0-mm-3D_firstorder_Range |
| 13 | log-sigma-1-0-mm-3D_firstorder_RobustMeanAbsoluteDeviation |
| 14 | log-sigma-1-0-mm-3D_firstorder_RootMeanSquared |
| 15 | log-sigma-1-0-mm-3D_firstorder_Skewness |
| 16 | log-sigma-1-0-mm-3D_firstorder_TotalEnergy |
| 17 | log-sigma-1-0-mm-3D_firstorder_Uniformity |
| 18 | log-sigma-1-0-mm-3D_firstorder_Variance |
| 19 | log-sigma-1-0-mm-3D_glcm_Autocorrelation |
| 20 | log-sigma-1-0-mm-3D_glcm_ClusterProminence |
| 21 | log-sigma-1-0-mm-3D_glcm_ClusterShade |
| 22 | log-sigma-1-0-mm-3D_glcm_ClusterTendency |
| 23 | log-sigma-1-0-mm-3D_glcm_Contrast |
| 24 | log-sigma-1-0-mm-3D_glcm_Correlation |
| 25 | log-sigma-1-0-mm-3D_glcm_DifferenceAverage |
| 26 | log-sigma-1-0-mm-3D_glcm_DifferenceEntropy |
| 27 | log-sigma-1-0-mm-3D_glcm_DifferenceVariance |
| 28 | log-sigma-1-0-mm-3D_glcm_Id |
| 29 | log-sigma-1-0-mm-3D_glcm_Idm |
| 30 | log-sigma-1-0-mm-3D_glcm_Idmn |
| 31 | log-sigma-1-0-mm-3D_glcm_Idn |
| 32 | log-sigma-1-0-mm-3D_glcm_Imc1 |
| 33 | log-sigma-1-0-mm-3D_glcm_Imc2 |
| 34 | log-sigma-1-0-mm-3D_glcm_InverseVariance |
| 35 | log-sigma-1-0-mm-3D_glcm_JointAverage |
| 36 | log-sigma-1-0-mm-3D_glcm_JointEnergy |
| 37 | log-sigma-1-0-mm-3D_glcm_JointEntropy |
| 38 | log-sigma-1-0-mm-3D_glcm_MaximumProbability |
| 39 | log-sigma-1-0-mm-3D_glcm_MCC |
| 40 | log-sigma-1-0-mm-3D_glcm_SumAverage |
| 41 | log-sigma-1-0-mm-3D_glcm_SumEntropy |
| 42 | log-sigma-1-0-mm-3D_glcm_SumSquares |
| 43 | log-sigma-1-0-mm-3D_gldm_DependenceEntropy |
| 44 | log-sigma-1-0-mm-3D_gldm_DependenceNonUniformity |
| 45 | log-sigma-1-0-mm-3D_gldm_DependenceNonUniformityNormalized |
| 46 | log-sigma-1-0-mm-3D_gldm_DependenceVariance |
| 47 | log-sigma-1-0-mm-3D_gldm_GrayLevelNonUniformity |
| 48 | log-sigma-1-0-mm-3D_gldm_GrayLevelVariance |
| 49 | log-sigma-1-0-mm-3D_gldm_HighGrayLevelEmphasis |
| 50 | log-sigma-1-0-mm-3D_gldm_LargeDependenceEmphasis |
| 51 | log-sigma-1-0-mm-3D_gldm_LargeDependenceHighGrayLevelEmphasis |
| 52 | log-sigma-1-0-mm-3D_gldm_LargeDependenceLowGrayLevelEmphasis |
| 53 | log-sigma-1-0-mm-3D_gldm_LowGrayLevelEmphasis |
| 54 | log-sigma-1-0-mm-3D_gldm_SmallDependenceEmphasis |
| 55 | log-sigma-1-0-mm-3D_gldm_SmallDependenceHighGrayLevelEmphasis |
| 56 | log-sigma-1-0-mm-3D_gldm_SmallDependenceLowGrayLevelEmphasis |
| 57 | log-sigma-1-0-mm-3D_glrlm_GrayLevelNonUniformity |
| 58 | log-sigma-1-0-mm-3D_glrlm_GrayLevelNonUniformityNormalized |
| 59 | log-sigma-1-0-mm-3D_glrlm_GrayLevelVariance |
| 60 | log-sigma-1-0-mm-3D_glrlm_HighGrayLevelRunEmphasis |
| 61 | log-sigma-1-0-mm-3D_glrlm_LongRunEmphasis |
| 62 | log-sigma-1-0-mm-3D_glrlm_LongRunHighGrayLevelEmphasis |
| 63 | log-sigma-1-0-mm-3D_glrlm_LongRunLowGrayLevelEmphasis |
| 64 | log-sigma-1-0-mm-3D_glrlm_LowGrayLevelRunEmphasis |
| 65 | log-sigma-1-0-mm-3D_glrlm_RunEntropy |
| 66 | log-sigma-1-0-mm-3D_glrlm_RunLengthNonUniformity |
| 67 | log-sigma-1-0-mm-3D_glrlm_RunLengthNonUniformityNormalized |
| 68 | log-sigma-1-0-mm-3D_glrlm_RunPercentage |
| 69 | log-sigma-1-0-mm-3D_glrlm_RunVariance |
| 70 | log-sigma-1-0-mm-3D_glrlm_ShortRunEmphasis |
| 71 | log-sigma-1-0-mm-3D_glrlm_ShortRunHighGrayLevelEmphasis |
| 72 | log-sigma-1-0-mm-3D_glrlm_ShortRunLowGrayLevelEmphasis |
| 73 | log-sigma-1-0-mm-3D_glszm_GrayLevelNonUniformity |
| 74 | log-sigma-1-0-mm-3D_glszm_GrayLevelNonUniformityNormalized |
| 75 | log-sigma-1-0-mm-3D_glszm_GrayLevelVariance |
| 76 | log-sigma-1-0-mm-3D_glszm_HighGrayLevelZoneEmphasis |
| 77 | log-sigma-1-0-mm-3D_glszm_LargeAreaEmphasis |
| 78 | log-sigma-1-0-mm-3D_glszm_LargeAreaHighGrayLevelEmphasis |
| 79 | log-sigma-1-0-mm-3D_glszm_LargeAreaLowGrayLevelEmphasis |
| 80 | log-sigma-1-0-mm-3D_glszm_LowGrayLevelZoneEmphasis |
| 81 | log-sigma-1-0-mm-3D_glszm_SizeZoneNonUniformity |
| 82 | log-sigma-1-0-mm-3D_glszm_SizeZoneNonUniformityNormalized |
| 83 | log-sigma-1-0-mm-3D_glszm_SmallAreaEmphasis |
| 84 | log-sigma-1-0-mm-3D_glszm_SmallAreaHighGrayLevelEmphasis |
| 85 | log-sigma-1-0-mm-3D_glszm_SmallAreaLowGrayLevelEmphasis |
| 86 | log-sigma-1-0-mm-3D_glszm_ZoneEntropy |
| 87 | log-sigma-1-0-mm-3D_glszm_ZonePercentage |
| 88 | log-sigma-1-0-mm-3D_glszm_ZoneVariance |
| 89 | log-sigma-1-0-mm-3D_ngtdm_Busyness |
| 90 | log-sigma-1-0-mm-3D_ngtdm_Coarseness |
| 91 | log-sigma-1-0-mm-3D_ngtdm_Complexity |
| 92 | log-sigma-1-0-mm-3D_ngtdm_Contrast |
| 93 | log-sigma-1-0-mm-3D_ngtdm_Strength |
| 94 | log-sigma-2-0-mm-3D_firstorder_10Percentile |
| 95 | log-sigma-2-0-mm-3D_firstorder_90Percentile |
| 96 | log-sigma-2-0-mm-3D_firstorder_Energy |
| 97 | log-sigma-2-0-mm-3D_firstorder_Entropy |
| 98 | log-sigma-2-0-mm-3D_firstorder_InterquartileRange |
| 99 | log-sigma-2-0-mm-3D_firstorder_Kurtosis |
| 100 | log-sigma-2-0-mm-3D_firstorder_Maximum |
| 101 | log-sigma-2-0-mm-3D_firstorder_Mean |
| 102 | log-sigma-2-0-mm-3D_firstorder_MeanAbsoluteDeviation |
| 103 | log-sigma-2-0-mm-3D_firstorder_Median |
| 104 | log-sigma-2-0-mm-3D_firstorder_Minimum |
| 105 | log-sigma-2-0-mm-3D_firstorder_Range |
| 106 | log-sigma-2-0-mm-3D_firstorder_RobustMeanAbsoluteDeviation |
| 107 | log-sigma-2-0-mm-3D_firstorder_RootMeanSquared |
| 108 | log-sigma-2-0-mm-3D_firstorder_Skewness |
| 109 | log-sigma-2-0-mm-3D_firstorder_TotalEnergy |
| 110 | log-sigma-2-0-mm-3D_firstorder_Uniformity |
| 111 | log-sigma-2-0-mm-3D_firstorder_Variance |
| 112 | log-sigma-2-0-mm-3D_glcm_Autocorrelation |
| 113 | log-sigma-2-0-mm-3D_glcm_ClusterProminence |
| 114 | log-sigma-2-0-mm-3D_glcm_ClusterShade |
| 115 | log-sigma-2-0-mm-3D_glcm_ClusterTendency |
| 116 | log-sigma-2-0-mm-3D_glcm_Contrast |
| 117 | log-sigma-2-0-mm-3D_glcm_Correlation |
| 118 | log-sigma-2-0-mm-3D_glcm_DifferenceAverage |
| 119 | log-sigma-2-0-mm-3D_glcm_DifferenceEntropy |
| 120 | log-sigma-2-0-mm-3D_glcm_DifferenceVariance |
| 121 | log-sigma-2-0-mm-3D_glcm_Id |
| 122 | log-sigma-2-0-mm-3D_glcm_Idm |
| 123 | log-sigma-2-0-mm-3D_glcm_Idmn |
| 124 | log-sigma-2-0-mm-3D_glcm_Idn |
| 125 | log-sigma-2-0-mm-3D_glcm_Imc1 |
| 126 | log-sigma-2-0-mm-3D_glcm_Imc2 |
| 127 | log-sigma-2-0-mm-3D_glcm_InverseVariance |
| 128 | log-sigma-2-0-mm-3D_glcm_JointAverage |
| 129 | log-sigma-2-0-mm-3D_glcm_JointEnergy |
| 130 | log-sigma-2-0-mm-3D_glcm_JointEntropy |
| 131 | log-sigma-2-0-mm-3D_glcm_MaximumProbability |
| 132 | log-sigma-2-0-mm-3D_glcm_MCC |
| 133 | log-sigma-2-0-mm-3D_glcm_SumAverage |
| 134 | log-sigma-2-0-mm-3D_glcm_SumEntropy |
| 135 | log-sigma-2-0-mm-3D_glcm_SumSquares |
| 136 | log-sigma-2-0-mm-3D_gldm_DependenceEntropy |
| 137 | log-sigma-2-0-mm-3D_gldm_DependenceNonUniformity |
| 138 | log-sigma-2-0-mm-3D_gldm_DependenceNonUniformityNormalized |
| 139 | log-sigma-2-0-mm-3D_gldm_DependenceVariance |
| 140 | log-sigma-2-0-mm-3D_gldm_GrayLevelNonUniformity |
| 141 | log-sigma-2-0-mm-3D_gldm_GrayLevelVariance |
| 142 | log-sigma-2-0-mm-3D_gldm_HighGrayLevelEmphasis |
| 143 | log-sigma-2-0-mm-3D_gldm_LargeDependenceEmphasis |
| 144 | log-sigma-2-0-mm-3D_gldm_LargeDependenceHighGrayLevelEmphasis |
| 145 | log-sigma-2-0-mm-3D_gldm_LargeDependenceLowGrayLevelEmphasis |
| 146 | log-sigma-2-0-mm-3D_gldm_LowGrayLevelEmphasis |
| 147 | log-sigma-2-0-mm-3D_gldm_SmallDependenceEmphasis |
| 148 | log-sigma-2-0-mm-3D_gldm_SmallDependenceHighGrayLevelEmphasis |
| 149 | log-sigma-2-0-mm-3D_gldm_SmallDependenceLowGrayLevelEmphasis |
| 150 | log-sigma-2-0-mm-3D_glrlm_GrayLevelNonUniformity |
| 151 | log-sigma-2-0-mm-3D_glrlm_GrayLevelNonUniformityNormalized |
| 152 | log-sigma-2-0-mm-3D_glrlm_GrayLevelVariance |
| 153 | log-sigma-2-0-mm-3D_glrlm_HighGrayLevelRunEmphasis |
| 154 | log-sigma-2-0-mm-3D_glrlm_LongRunEmphasis |
| 155 | log-sigma-2-0-mm-3D_glrlm_LongRunHighGrayLevelEmphasis |
| 156 | log-sigma-2-0-mm-3D_glrlm_LongRunLowGrayLevelEmphasis |
| 157 | log-sigma-2-0-mm-3D_glrlm_LowGrayLevelRunEmphasis |
| 158 | log-sigma-2-0-mm-3D_glrlm_RunEntropy |
| 159 | log-sigma-2-0-mm-3D_glrlm_RunLengthNonUniformity |
| 160 | log-sigma-2-0-mm-3D_glrlm_RunLengthNonUniformityNormalized |
| 161 | log-sigma-2-0-mm-3D_glrlm_RunPercentage |
| 162 | log-sigma-2-0-mm-3D_glrlm_RunVariance |
| 163 | log-sigma-2-0-mm-3D_glrlm_ShortRunEmphasis |
| 164 | log-sigma-2-0-mm-3D_glrlm_ShortRunHighGrayLevelEmphasis |
| 165 | log-sigma-2-0-mm-3D_glrlm_ShortRunLowGrayLevelEmphasis |
| 166 | log-sigma-2-0-mm-3D_glszm_GrayLevelNonUniformity |
| 167 | log-sigma-2-0-mm-3D_glszm_GrayLevelNonUniformityNormalized |
| 168 | log-sigma-2-0-mm-3D_glszm_GrayLevelVariance |
| 169 | log-sigma-2-0-mm-3D_glszm_HighGrayLevelZoneEmphasis |
| 170 | log-sigma-2-0-mm-3D_glszm_LargeAreaEmphasis |
| 171 | log-sigma-2-0-mm-3D_glszm_LargeAreaHighGrayLevelEmphasis |
| 172 | log-sigma-2-0-mm-3D_glszm_LargeAreaLowGrayLevelEmphasis |
| 173 | log-sigma-2-0-mm-3D_glszm_LowGrayLevelZoneEmphasis |
| 174 | log-sigma-2-0-mm-3D_glszm_SizeZoneNonUniformity |
| 175 | log-sigma-2-0-mm-3D_glszm_SizeZoneNonUniformityNormalized |
| 176 | log-sigma-2-0-mm-3D_glszm_SmallAreaEmphasis |
| 177 | log-sigma-2-0-mm-3D_glszm_SmallAreaHighGrayLevelEmphasis |
| 178 | log-sigma-2-0-mm-3D_glszm_SmallAreaLowGrayLevelEmphasis |
| 179 | log-sigma-2-0-mm-3D_glszm_ZoneEntropy |
| 180 | log-sigma-2-0-mm-3D_glszm_ZonePercentage |
| 181 | log-sigma-2-0-mm-3D_glszm_ZoneVariance |
| 182 | log-sigma-2-0-mm-3D_ngtdm_Busyness |
| 183 | log-sigma-2-0-mm-3D_ngtdm_Coarseness |
| 184 | log-sigma-2-0-mm-3D_ngtdm_Complexity |
| 185 | log-sigma-2-0-mm-3D_ngtdm_Contrast |
| 186 | log-sigma-2-0-mm-3D_ngtdm_Strength |
| 187 | log-sigma-3-0-mm-3D_firstorder_10Percentile |
| 188 | log-sigma-3-0-mm-3D_firstorder_90Percentile |
| 189 | log-sigma-3-0-mm-3D_firstorder_Energy |
| 190 | log-sigma-3-0-mm-3D_firstorder_Entropy |
| 191 | log-sigma-3-0-mm-3D_firstorder_InterquartileRange |
| 192 | log-sigma-3-0-mm-3D_firstorder_Kurtosis |
| 193 | log-sigma-3-0-mm-3D_firstorder_Maximum |
| 194 | log-sigma-3-0-mm-3D_firstorder_Mean |
| 195 | log-sigma-3-0-mm-3D_firstorder_MeanAbsoluteDeviation |
| 196 | log-sigma-3-0-mm-3D_firstorder_Median |
| 197 | log-sigma-3-0-mm-3D_firstorder_Minimum |
| 198 | log-sigma-3-0-mm-3D_firstorder_Range |
| 199 | log-sigma-3-0-mm-3D_firstorder_RobustMeanAbsoluteDeviation |
| 200 | log-sigma-3-0-mm-3D_firstorder_RootMeanSquared |
| 201 | log-sigma-3-0-mm-3D_firstorder_Skewness |
| 202 | log-sigma-3-0-mm-3D_firstorder_TotalEnergy |
| 203 | log-sigma-3-0-mm-3D_firstorder_Uniformity |
| 204 | log-sigma-3-0-mm-3D_firstorder_Variance |
| 205 | log-sigma-3-0-mm-3D_glcm_Autocorrelation |
| 206 | log-sigma-3-0-mm-3D_glcm_ClusterProminence |
| 207 | log-sigma-3-0-mm-3D_glcm_ClusterShade |
| 208 | log-sigma-3-0-mm-3D_glcm_ClusterTendency |
| 209 | log-sigma-3-0-mm-3D_glcm_Contrast |
| 210 | log-sigma-3-0-mm-3D_glcm_Correlation |
| 211 | log-sigma-3-0-mm-3D_glcm_DifferenceAverage |
| 212 | log-sigma-3-0-mm-3D_glcm_DifferenceEntropy |
| 213 | log-sigma-3-0-mm-3D_glcm_DifferenceVariance |
| 214 | log-sigma-3-0-mm-3D_glcm_Id |
| 215 | log-sigma-3-0-mm-3D_glcm_Idm |
| 216 | log-sigma-3-0-mm-3D_glcm_Idmn |
| 217 | log-sigma-3-0-mm-3D_glcm_Idn |
| 218 | log-sigma-3-0-mm-3D_glcm_Imc1 |
| 219 | log-sigma-3-0-mm-3D_glcm_Imc2 |
| 220 | log-sigma-3-0-mm-3D_glcm_InverseVariance |
| 221 | log-sigma-3-0-mm-3D_glcm_JointAverage |
| 222 | log-sigma-3-0-mm-3D_glcm_JointEnergy |
| 223 | log-sigma-3-0-mm-3D_glcm_JointEntropy |
| 224 | log-sigma-3-0-mm-3D_glcm_MaximumProbability |
| 225 | log-sigma-3-0-mm-3D_glcm_MCC |
| 226 | log-sigma-3-0-mm-3D_glcm_SumAverage |
| 227 | log-sigma-3-0-mm-3D_glcm_SumEntropy |
| 228 | log-sigma-3-0-mm-3D_glcm_SumSquares |
| 229 | log-sigma-3-0-mm-3D_gldm_DependenceEntropy |
| 230 | log-sigma-3-0-mm-3D_gldm_DependenceNonUniformity |
| 231 | log-sigma-3-0-mm-3D_gldm_DependenceNonUniformityNormalized |
| 232 | log-sigma-3-0-mm-3D_gldm_DependenceVariance |
| 233 | log-sigma-3-0-mm-3D_gldm_GrayLevelNonUniformity |
| 234 | log-sigma-3-0-mm-3D_gldm_GrayLevelVariance |
| 235 | log-sigma-3-0-mm-3D_gldm_HighGrayLevelEmphasis |
| 236 | log-sigma-3-0-mm-3D_gldm_LargeDependenceEmphasis |
| 237 | log-sigma-3-0-mm-3D_gldm_LargeDependenceHighGrayLevelEmphasis |
| 238 | log-sigma-3-0-mm-3D_gldm_LargeDependenceLowGrayLevelEmphasis |
| 239 | log-sigma-3-0-mm-3D_gldm_LowGrayLevelEmphasis |
| 240 | log-sigma-3-0-mm-3D_gldm_SmallDependenceEmphasis |
| 241 | log-sigma-3-0-mm-3D_gldm_SmallDependenceHighGrayLevelEmphasis |
| 242 | log-sigma-3-0-mm-3D_gldm_SmallDependenceLowGrayLevelEmphasis |
| 243 | log-sigma-3-0-mm-3D_glrlm_GrayLevelNonUniformity |
| 244 | log-sigma-3-0-mm-3D_glrlm_GrayLevelNonUniformityNormalized |
| 245 | log-sigma-3-0-mm-3D_glrlm_GrayLevelVariance |
| 246 | log-sigma-3-0-mm-3D_glrlm_HighGrayLevelRunEmphasis |
| 247 | log-sigma-3-0-mm-3D_glrlm_LongRunEmphasis |
| 248 | log-sigma-3-0-mm-3D_glrlm_LongRunHighGrayLevelEmphasis |
| 249 | log-sigma-3-0-mm-3D_glrlm_LongRunLowGrayLevelEmphasis |
| 250 | log-sigma-3-0-mm-3D_glrlm_LowGrayLevelRunEmphasis |
| 251 | log-sigma-3-0-mm-3D_glrlm_RunEntropy |
| 252 | log-sigma-3-0-mm-3D_glrlm_RunLengthNonUniformity |
| 253 | log-sigma-3-0-mm-3D_glrlm_RunLengthNonUniformityNormalized |
| 254 | log-sigma-3-0-mm-3D_glrlm_RunPercentage |
| 255 | log-sigma-3-0-mm-3D_glrlm_RunVariance |
| 256 | log-sigma-3-0-mm-3D_glrlm_ShortRunEmphasis |
| 257 | log-sigma-3-0-mm-3D_glrlm_ShortRunHighGrayLevelEmphasis |
| 258 | log-sigma-3-0-mm-3D_glrlm_ShortRunLowGrayLevelEmphasis |
| 259 | log-sigma-3-0-mm-3D_glszm_GrayLevelNonUniformity |
| 260 | log-sigma-3-0-mm-3D_glszm_GrayLevelNonUniformityNormalized |
| 261 | log-sigma-3-0-mm-3D_glszm_GrayLevelVariance |
| 262 | log-sigma-3-0-mm-3D_glszm_HighGrayLevelZoneEmphasis |
| 263 | log-sigma-3-0-mm-3D_glszm_LargeAreaEmphasis |
| 264 | log-sigma-3-0-mm-3D_glszm_LargeAreaHighGrayLevelEmphasis |
| 265 | log-sigma-3-0-mm-3D_glszm_LargeAreaLowGrayLevelEmphasis |
| 266 | log-sigma-3-0-mm-3D_glszm_LowGrayLevelZoneEmphasis |
| 267 | log-sigma-3-0-mm-3D_glszm_SizeZoneNonUniformity |
| 268 | log-sigma-3-0-mm-3D_glszm_SizeZoneNonUniformityNormalized |
| 269 | log-sigma-3-0-mm-3D_glszm_SmallAreaEmphasis |
| 270 | log-sigma-3-0-mm-3D_glszm_SmallAreaHighGrayLevelEmphasis |
| 271 | log-sigma-3-0-mm-3D_glszm_SmallAreaLowGrayLevelEmphasis |
| 272 | log-sigma-3-0-mm-3D_glszm_ZoneEntropy |
| 273 | log-sigma-3-0-mm-3D_glszm_ZonePercentage |
| 274 | log-sigma-3-0-mm-3D_glszm_ZoneVariance |
| 275 | log-sigma-3-0-mm-3D_ngtdm_Busyness |
| 276 | log-sigma-3-0-mm-3D_ngtdm_Coarseness |
| 277 | log-sigma-3-0-mm-3D_ngtdm_Complexity |
| 278 | log-sigma-3-0-mm-3D_ngtdm_Contrast |
| 279 | log-sigma-3-0-mm-3D_ngtdm_Strength |
| 280 | log-sigma-4-0-mm-3D_firstorder_10Percentile |
| 281 | log-sigma-4-0-mm-3D_firstorder_90Percentile |
| 282 | log-sigma-4-0-mm-3D_firstorder_Energy |
| 283 | log-sigma-4-0-mm-3D_firstorder_Entropy |
| 284 | log-sigma-4-0-mm-3D_firstorder_InterquartileRange |
| 285 | log-sigma-4-0-mm-3D_firstorder_Kurtosis |
| 286 | log-sigma-4-0-mm-3D_firstorder_Maximum |
| 287 | log-sigma-4-0-mm-3D_firstorder_Mean |
| 288 | log-sigma-4-0-mm-3D_firstorder_MeanAbsoluteDeviation |
| 289 | log-sigma-4-0-mm-3D_firstorder_Median |
| 290 | log-sigma-4-0-mm-3D_firstorder_Minimum |
| 291 | log-sigma-4-0-mm-3D_firstorder_Range |
| 292 | log-sigma-4-0-mm-3D_firstorder_RobustMeanAbsoluteDeviation |
| 293 | log-sigma-4-0-mm-3D_firstorder_RootMeanSquared |
| 294 | log-sigma-4-0-mm-3D_firstorder_Skewness |
| 295 | log-sigma-4-0-mm-3D_firstorder_TotalEnergy |
| 296 | log-sigma-4-0-mm-3D_firstorder_Uniformity |
| 297 | log-sigma-4-0-mm-3D_firstorder_Variance |
| 298 | log-sigma-4-0-mm-3D_glcm_Autocorrelation |
| 299 | log-sigma-4-0-mm-3D_glcm_ClusterProminence |
| 300 | log-sigma-4-0-mm-3D_glcm_ClusterShade |
| 301 | log-sigma-4-0-mm-3D_glcm_ClusterTendency |
| 302 | log-sigma-4-0-mm-3D_glcm_Contrast |
| 303 | log-sigma-4-0-mm-3D_glcm_Correlation |
| 304 | log-sigma-4-0-mm-3D_glcm_DifferenceAverage |
| 305 | log-sigma-4-0-mm-3D_glcm_DifferenceEntropy |
| 306 | log-sigma-4-0-mm-3D_glcm_DifferenceVariance |
| 307 | log-sigma-4-0-mm-3D_glcm_Id |
| 308 | log-sigma-4-0-mm-3D_glcm_Idm |
| 309 | log-sigma-4-0-mm-3D_glcm_Idmn |
| 310 | log-sigma-4-0-mm-3D_glcm_Idn |
| 311 | log-sigma-4-0-mm-3D_glcm_Imc1 |
| 312 | log-sigma-4-0-mm-3D_glcm_Imc2 |
| 313 | log-sigma-4-0-mm-3D_glcm_InverseVariance |
| 314 | log-sigma-4-0-mm-3D_glcm_JointAverage |
| 315 | log-sigma-4-0-mm-3D_glcm_JointEnergy |
| 316 | log-sigma-4-0-mm-3D_glcm_JointEntropy |
| 317 | log-sigma-4-0-mm-3D_glcm_MaximumProbability |
| 318 | log-sigma-4-0-mm-3D_glcm_MCC |
| 319 | log-sigma-4-0-mm-3D_glcm_SumAverage |
| 320 | log-sigma-4-0-mm-3D_glcm_SumEntropy |
| 321 | log-sigma-4-0-mm-3D_glcm_SumSquares |
| 322 | log-sigma-4-0-mm-3D_gldm_DependenceEntropy |
| 323 | log-sigma-4-0-mm-3D_gldm_DependenceNonUniformity |
| 324 | log-sigma-4-0-mm-3D_gldm_DependenceNonUniformityNormalized |
| 325 | log-sigma-4-0-mm-3D_gldm_DependenceVariance |
| 326 | log-sigma-4-0-mm-3D_gldm_GrayLevelNonUniformity |
| 327 | log-sigma-4-0-mm-3D_gldm_GrayLevelVariance |
| 328 | log-sigma-4-0-mm-3D_gldm_HighGrayLevelEmphasis |
| 329 | log-sigma-4-0-mm-3D_gldm_LargeDependenceEmphasis |
| 330 | log-sigma-4-0-mm-3D_gldm_LargeDependenceHighGrayLevelEmphasis |
| 331 | log-sigma-4-0-mm-3D_gldm_LargeDependenceLowGrayLevelEmphasis |
| 332 | log-sigma-4-0-mm-3D_gldm_LowGrayLevelEmphasis |
| 333 | log-sigma-4-0-mm-3D_gldm_SmallDependenceEmphasis |
| 334 | log-sigma-4-0-mm-3D_gldm_SmallDependenceHighGrayLevelEmphasis |
| 335 | log-sigma-4-0-mm-3D_gldm_SmallDependenceLowGrayLevelEmphasis |
| 336 | log-sigma-4-0-mm-3D_glrlm_GrayLevelNonUniformity |
| 337 | log-sigma-4-0-mm-3D_glrlm_GrayLevelNonUniformityNormalized |
| 338 | log-sigma-4-0-mm-3D_glrlm_GrayLevelVariance |
| 339 | log-sigma-4-0-mm-3D_glrlm_HighGrayLevelRunEmphasis |
| 340 | log-sigma-4-0-mm-3D_glrlm_LongRunEmphasis |
| 341 | log-sigma-4-0-mm-3D_glrlm_LongRunHighGrayLevelEmphasis |
| 342 | log-sigma-4-0-mm-3D_glrlm_LongRunLowGrayLevelEmphasis |
| 343 | log-sigma-4-0-mm-3D_glrlm_LowGrayLevelRunEmphasis |
| 344 | log-sigma-4-0-mm-3D_glrlm_RunEntropy |
| 345 | log-sigma-4-0-mm-3D_glrlm_RunLengthNonUniformity |
| 346 | log-sigma-4-0-mm-3D_glrlm_RunLengthNonUniformityNormalized |
| 347 | log-sigma-4-0-mm-3D_glrlm_RunPercentage |
| 348 | log-sigma-4-0-mm-3D_glrlm_RunVariance |
| 349 | log-sigma-4-0-mm-3D_glrlm_ShortRunEmphasis |
| 350 | log-sigma-4-0-mm-3D_glrlm_ShortRunHighGrayLevelEmphasis |
| 351 | log-sigma-4-0-mm-3D_glrlm_ShortRunLowGrayLevelEmphasis |
| 352 | log-sigma-4-0-mm-3D_glszm_GrayLevelNonUniformity |
| 353 | log-sigma-4-0-mm-3D_glszm_GrayLevelNonUniformityNormalized |
| 354 | log-sigma-4-0-mm-3D_glszm_GrayLevelVariance |
| 355 | log-sigma-4-0-mm-3D_glszm_HighGrayLevelZoneEmphasis |
| 356 | log-sigma-4-0-mm-3D_glszm_LargeAreaEmphasis |
| 357 | log-sigma-4-0-mm-3D_glszm_LargeAreaHighGrayLevelEmphasis |
| 358 | log-sigma-4-0-mm-3D_glszm_LargeAreaLowGrayLevelEmphasis |
| 359 | log-sigma-4-0-mm-3D_glszm_LowGrayLevelZoneEmphasis |
| 360 | log-sigma-4-0-mm-3D_glszm_SizeZoneNonUniformity |
| 361 | log-sigma-4-0-mm-3D_glszm_SizeZoneNonUniformityNormalized |
| 362 | log-sigma-4-0-mm-3D_glszm_SmallAreaEmphasis |
| 363 | log-sigma-4-0-mm-3D_glszm_SmallAreaHighGrayLevelEmphasis |
| 364 | log-sigma-4-0-mm-3D_glszm_SmallAreaLowGrayLevelEmphasis |
| 365 | log-sigma-4-0-mm-3D_glszm_ZoneEntropy |
| 366 | log-sigma-4-0-mm-3D_glszm_ZonePercentage |
| 367 | log-sigma-4-0-mm-3D_glszm_ZoneVariance |
| 368 | log-sigma-4-0-mm-3D_ngtdm_Busyness |
| 369 | log-sigma-4-0-mm-3D_ngtdm_Coarseness |
| 370 | log-sigma-4-0-mm-3D_ngtdm_Complexity |
| 371 | log-sigma-4-0-mm-3D_ngtdm_Contrast |
| 372 | log-sigma-4-0-mm-3D_ngtdm_Strength |
| 373 | log-sigma-5-0-mm-3D_firstorder_10Percentile |
| 374 | log-sigma-5-0-mm-3D_firstorder_90Percentile |
| 375 | log-sigma-5-0-mm-3D_firstorder_Energy |
| 376 | log-sigma-5-0-mm-3D_firstorder_Entropy |
| 377 | log-sigma-5-0-mm-3D_firstorder_InterquartileRange |
| 378 | log-sigma-5-0-mm-3D_firstorder_Kurtosis |
| 379 | log-sigma-5-0-mm-3D_firstorder_Maximum |
| 380 | log-sigma-5-0-mm-3D_firstorder_Mean |
| 381 | log-sigma-5-0-mm-3D_firstorder_MeanAbsoluteDeviation |
| 382 | log-sigma-5-0-mm-3D_firstorder_Median |
| 383 | log-sigma-5-0-mm-3D_firstorder_Minimum |
| 384 | log-sigma-5-0-mm-3D_firstorder_Range |
| 385 | log-sigma-5-0-mm-3D_firstorder_RobustMeanAbsoluteDeviation |
| 386 | log-sigma-5-0-mm-3D_firstorder_RootMeanSquared |
| 387 | log-sigma-5-0-mm-3D_firstorder_Skewness |
| 388 | log-sigma-5-0-mm-3D_firstorder_TotalEnergy |
| 389 | log-sigma-5-0-mm-3D_firstorder_Uniformity |
| 390 | log-sigma-5-0-mm-3D_firstorder_Variance |
| 391 | log-sigma-5-0-mm-3D_glcm_Autocorrelation |
| 392 | log-sigma-5-0-mm-3D_glcm_ClusterProminence |
| 393 | log-sigma-5-0-mm-3D_glcm_ClusterShade |
| 394 | log-sigma-5-0-mm-3D_glcm_ClusterTendency |
| 395 | log-sigma-5-0-mm-3D_glcm_Contrast |
| 396 | log-sigma-5-0-mm-3D_glcm_Correlation |
| 397 | log-sigma-5-0-mm-3D_glcm_DifferenceAverage |
| 398 | log-sigma-5-0-mm-3D_glcm_DifferenceEntropy |
| 399 | log-sigma-5-0-mm-3D_glcm_DifferenceVariance |
| 400 | log-sigma-5-0-mm-3D_glcm_Id |
| 401 | log-sigma-5-0-mm-3D_glcm_Idm |
| 402 | log-sigma-5-0-mm-3D_glcm_Idmn |
| 403 | log-sigma-5-0-mm-3D_glcm_Idn |
| 404 | log-sigma-5-0-mm-3D_glcm_Imc1 |
| 405 | log-sigma-5-0-mm-3D_glcm_Imc2 |
| 406 | log-sigma-5-0-mm-3D_glcm_InverseVariance |
| 407 | log-sigma-5-0-mm-3D_glcm_JointAverage |
| 408 | log-sigma-5-0-mm-3D_glcm_JointEnergy |
| 409 | log-sigma-5-0-mm-3D_glcm_JointEntropy |
| 410 | log-sigma-5-0-mm-3D_glcm_MaximumProbability |
| 411 | log-sigma-5-0-mm-3D_glcm_MCC |
| 412 | log-sigma-5-0-mm-3D_glcm_SumAverage |
| 413 | log-sigma-5-0-mm-3D_glcm_SumEntropy |
| 414 | log-sigma-5-0-mm-3D_glcm_SumSquares |
| 415 | log-sigma-5-0-mm-3D_gldm_DependenceEntropy |
| 416 | log-sigma-5-0-mm-3D_gldm_DependenceNonUniformity |
| 417 | log-sigma-5-0-mm-3D_gldm_DependenceNonUniformityNormalized |
| 418 | log-sigma-5-0-mm-3D_gldm_DependenceVariance |
| 419 | log-sigma-5-0-mm-3D_gldm_GrayLevelNonUniformity |
| 420 | log-sigma-5-0-mm-3D_gldm_GrayLevelVariance |
| 421 | log-sigma-5-0-mm-3D_gldm_HighGrayLevelEmphasis |
| 422 | log-sigma-5-0-mm-3D_gldm_LargeDependenceEmphasis |
| 423 | log-sigma-5-0-mm-3D_gldm_LargeDependenceHighGrayLevelEmphasis |
| 424 | log-sigma-5-0-mm-3D_gldm_LargeDependenceLowGrayLevelEmphasis |
| 425 | log-sigma-5-0-mm-3D_gldm_LowGrayLevelEmphasis |
| 426 | log-sigma-5-0-mm-3D_gldm_SmallDependenceEmphasis |
| 427 | log-sigma-5-0-mm-3D_gldm_SmallDependenceHighGrayLevelEmphasis |
| 428 | log-sigma-5-0-mm-3D_gldm_SmallDependenceLowGrayLevelEmphasis |
| 429 | log-sigma-5-0-mm-3D_glrlm_GrayLevelNonUniformity |
| 430 | log-sigma-5-0-mm-3D_glrlm_GrayLevelNonUniformityNormalized |
| 431 | log-sigma-5-0-mm-3D_glrlm_GrayLevelVariance |
| 432 | log-sigma-5-0-mm-3D_glrlm_HighGrayLevelRunEmphasis |
| 433 | log-sigma-5-0-mm-3D_glrlm_LongRunEmphasis |
| 434 | log-sigma-5-0-mm-3D_glrlm_LongRunHighGrayLevelEmphasis |
| 435 | log-sigma-5-0-mm-3D_glrlm_LongRunLowGrayLevelEmphasis |
| 436 | log-sigma-5-0-mm-3D_glrlm_LowGrayLevelRunEmphasis |
| 437 | log-sigma-5-0-mm-3D_glrlm_RunEntropy |
| 438 | log-sigma-5-0-mm-3D_glrlm_RunLengthNonUniformity |
| 439 | log-sigma-5-0-mm-3D_glrlm_RunLengthNonUniformityNormalized |
| 440 | log-sigma-5-0-mm-3D_glrlm_RunPercentage |
| 441 | log-sigma-5-0-mm-3D_glrlm_RunVariance |
| 442 | log-sigma-5-0-mm-3D_glrlm_ShortRunEmphasis |
| 443 | log-sigma-5-0-mm-3D_glrlm_ShortRunHighGrayLevelEmphasis |
| 444 | log-sigma-5-0-mm-3D_glrlm_ShortRunLowGrayLevelEmphasis |
| 445 | log-sigma-5-0-mm-3D_glszm_GrayLevelNonUniformity |
| 446 | log-sigma-5-0-mm-3D_glszm_GrayLevelNonUniformityNormalized |
| 447 | log-sigma-5-0-mm-3D_glszm_GrayLevelVariance |
| 448 | log-sigma-5-0-mm-3D_glszm_HighGrayLevelZoneEmphasis |
| 449 | log-sigma-5-0-mm-3D_glszm_LargeAreaEmphasis |
| 450 | log-sigma-5-0-mm-3D_glszm_LargeAreaHighGrayLevelEmphasis |
| 451 | log-sigma-5-0-mm-3D_glszm_LargeAreaLowGrayLevelEmphasis |
| 452 | log-sigma-5-0-mm-3D_glszm_LowGrayLevelZoneEmphasis |
| 453 | log-sigma-5-0-mm-3D_glszm_SizeZoneNonUniformity |
| 454 | log-sigma-5-0-mm-3D_glszm_SizeZoneNonUniformityNormalized |
| 455 | log-sigma-5-0-mm-3D_glszm_SmallAreaEmphasis |
| 456 | log-sigma-5-0-mm-3D_glszm_SmallAreaHighGrayLevelEmphasis |
| 457 | log-sigma-5-0-mm-3D_glszm_SmallAreaLowGrayLevelEmphasis |
| 458 | log-sigma-5-0-mm-3D_glszm_ZoneEntropy |
| 459 | log-sigma-5-0-mm-3D_glszm_ZonePercentage |
| 460 | log-sigma-5-0-mm-3D_glszm_ZoneVariance |
| 461 | log-sigma-5-0-mm-3D_ngtdm_Busyness |
| 462 | log-sigma-5-0-mm-3D_ngtdm_Coarseness |
| 463 | log-sigma-5-0-mm-3D_ngtdm_Complexity |
| 464 | log-sigma-5-0-mm-3D_ngtdm_Contrast |
| 465 | log-sigma-5-0-mm-3D_ngtdm_Strength |
| 466 | original_firstorder_10Percentile |
| 467 | original_firstorder_90Percentile |
| 468 | original_firstorder_Energy |
| 469 | original_firstorder_Entropy |
| 470 | original_firstorder_InterquartileRange |
| 471 | original_firstorder_Kurtosis |
| 472 | original_firstorder_Maximum |
| 473 | original_firstorder_Mean |
| 474 | original_firstorder_MeanAbsoluteDeviation |
| 475 | original_firstorder_Median |
| 476 | original_firstorder_Minimum |
| 477 | original_firstorder_Range |
| 478 | original_firstorder_RobustMeanAbsoluteDeviation |
| 479 | original_firstorder_RootMeanSquared |
| 480 | original_firstorder_Skewness |
| 481 | original_firstorder_TotalEnergy |
| 482 | original_firstorder_Uniformity |
| 483 | original_firstorder_Variance |
| 484 | original_glcm_Autocorrelation |
| 485 | original_glcm_ClusterProminence |
| 486 | original_glcm_ClusterShade |
| 487 | original_glcm_ClusterTendency |
| 488 | original_glcm_Contrast |
| 489 | original_glcm_Correlation |
| 490 | original_glcm_DifferenceAverage |
| 491 | original_glcm_DifferenceEntropy |
| 492 | original_glcm_DifferenceVariance |
| 493 | original_glcm_Id |
| 494 | original_glcm_Idm |
| 495 | original_glcm_Idmn |
| 496 | original_glcm_Idn |
| 497 | original_glcm_Imc1 |
| 498 | original_glcm_Imc2 |
| 499 | original_glcm_InverseVariance |
| 500 | original_glcm_JointAverage |
| 501 | original_glcm_JointEnergy |
| 502 | original_glcm_JointEntropy |
| 503 | original_glcm_MaximumProbability |
| 504 | original_glcm_MCC |
| 505 | original_glcm_SumAverage |
| 506 | original_glcm_SumEntropy |
| 507 | original_glcm_SumSquares |
| 508 | original_gldm_DependenceEntropy |
| 509 | original_gldm_DependenceNonUniformity |
| 510 | original_gldm_DependenceNonUniformityNormalized |
| 511 | original_gldm_DependenceVariance |
| 512 | original_gldm_GrayLevelNonUniformity |
| 513 | original_gldm_GrayLevelVariance |
| 514 | original_gldm_HighGrayLevelEmphasis |
| 515 | original_gldm_LargeDependenceEmphasis |
| 516 | original_gldm_LargeDependenceHighGrayLevelEmphasis |
| 517 | original_gldm_LargeDependenceLowGrayLevelEmphasis |
| 518 | original_gldm_LowGrayLevelEmphasis |
| 519 | original_gldm_SmallDependenceEmphasis |
| 520 | original_gldm_SmallDependenceHighGrayLevelEmphasis |
| 521 | original_gldm_SmallDependenceLowGrayLevelEmphasis |
| 522 | original_glrlm_GrayLevelNonUniformity |
| 523 | original_glrlm_GrayLevelNonUniformityNormalized |
| 524 | original_glrlm_GrayLevelVariance |
| 525 | original_glrlm_HighGrayLevelRunEmphasis |
| 526 | original_glrlm_LongRunEmphasis |
| 527 | original_glrlm_LongRunHighGrayLevelEmphasis |
| 528 | original_glrlm_LongRunLowGrayLevelEmphasis |
| 529 | original_glrlm_LowGrayLevelRunEmphasis |
| 530 | original_glrlm_RunEntropy |
| 531 | original_glrlm_RunLengthNonUniformity |
| 532 | original_glrlm_RunLengthNonUniformityNormalized |
| 533 | original_glrlm_RunPercentage |
| 534 | original_glrlm_RunVariance |
| 535 | original_glrlm_ShortRunEmphasis |
| 536 | original_glrlm_ShortRunHighGrayLevelEmphasis |
| 537 | original_glrlm_ShortRunLowGrayLevelEmphasis |
| 538 | original_glszm_GrayLevelNonUniformity |
| 539 | original_glszm_GrayLevelNonUniformityNormalized |
| 540 | original_glszm_GrayLevelVariance |
| 541 | original_glszm_HighGrayLevelZoneEmphasis |
| 542 | original_glszm_LargeAreaEmphasis |
| 543 | original_glszm_LargeAreaHighGrayLevelEmphasis |
| 544 | original_glszm_LargeAreaLowGrayLevelEmphasis |
| 545 | original_glszm_LowGrayLevelZoneEmphasis |
| 546 | original_glszm_SizeZoneNonUniformity |
| 547 | original_glszm_SizeZoneNonUniformityNormalized |
| 548 | original_glszm_SmallAreaEmphasis |
| 549 | original_glszm_SmallAreaHighGrayLevelEmphasis |
| 550 | original_glszm_SmallAreaLowGrayLevelEmphasis |
| 551 | original_glszm_ZoneEntropy |
| 552 | original_glszm_ZonePercentage |
| 553 | original_glszm_ZoneVariance |
| 554 | original_ngtdm_Busyness |
| 555 | original_ngtdm_Coarseness |
| 556 | original_ngtdm_Complexity |
| 557 | original_ngtdm_Contrast |
| 558 | original_ngtdm_Strength |
| 559 | original_shape_Elongation |
| 560 | original_shape_Flatness |
| 561 | original_shape_LeastAxisLength |
| 562 | original_shape_MajorAxisLength |
| 563 | original_shape_Maximum2DDiameterColumn |
| 564 | original_shape_Maximum2DDiameterRow |
| 565 | original_shape_Maximum2DDiameterSlice |
| 566 | original_shape_Maximum3DDiameter |
| 567 | original_shape_MeshVolume |
| 568 | original_shape_MinorAxisLength |
| 569 | original_shape_Sphericity |
| 570 | original_shape_SurfaceArea |
| 571 | original_shape_SurfaceVolumeRatio |
| 572 | original_shape_VoxelVolume |
| 573 | wavelet-HHH_firstorder_10Percentile |
| 574 | wavelet-HHH_firstorder_90Percentile |
| 575 | wavelet-HHH_firstorder_Energy |
| 576 | wavelet-HHH_firstorder_Entropy |
| 577 | wavelet-HHH_firstorder_InterquartileRange |
| 578 | wavelet-HHH_firstorder_Kurtosis |
| 579 | wavelet-HHH_firstorder_Maximum |
| 580 | wavelet-HHH_firstorder_Mean |
| 581 | wavelet-HHH_firstorder_MeanAbsoluteDeviation |
| 582 | wavelet-HHH_firstorder_Median |
| 583 | wavelet-HHH_firstorder_Minimum |
| 584 | wavelet-HHH_firstorder_Range |
| 585 | wavelet-HHH_firstorder_RobustMeanAbsoluteDeviation |
| 586 | wavelet-HHH_firstorder_RootMeanSquared |
| 587 | wavelet-HHH_firstorder_Skewness |
| 588 | wavelet-HHH_firstorder_TotalEnergy |
| 589 | wavelet-HHH_firstorder_Uniformity |
| 590 | wavelet-HHH_firstorder_Variance |
| 591 | wavelet-HHH_glcm_Autocorrelation |
| 592 | wavelet-HHH_glcm_ClusterProminence |
| 593 | wavelet-HHH_glcm_ClusterShade |
| 594 | wavelet-HHH_glcm_ClusterTendency |
| 595 | wavelet-HHH_glcm_Contrast |
| 596 | wavelet-HHH_glcm_Correlation |
| 597 | wavelet-HHH_glcm_DifferenceAverage |
| 598 | wavelet-HHH_glcm_DifferenceEntropy |
| 599 | wavelet-HHH_glcm_DifferenceVariance |
| 600 | wavelet-HHH_glcm_Id |
| 601 | wavelet-HHH_glcm_Idm |
| 602 | wavelet-HHH_glcm_Idmn |
| 603 | wavelet-HHH_glcm_Idn |
| 604 | wavelet-HHH_glcm_Imc1 |
| 605 | wavelet-HHH_glcm_Imc2 |
| 606 | wavelet-HHH_glcm_InverseVariance |
| 607 | wavelet-HHH_glcm_JointAverage |
| 608 | wavelet-HHH_glcm_JointEnergy |
| 609 | wavelet-HHH_glcm_JointEntropy |
| 610 | wavelet-HHH_glcm_MaximumProbability |
| 611 | wavelet-HHH_glcm_MCC |
| 612 | wavelet-HHH_glcm_SumAverage |
| 613 | wavelet-HHH_glcm_SumEntropy |
| 614 | wavelet-HHH_glcm_SumSquares |
| 615 | wavelet-HHH_gldm_DependenceEntropy |
| 616 | wavelet-HHH_gldm_DependenceNonUniformity |
| 617 | wavelet-HHH_gldm_DependenceNonUniformityNormalized |
| 618 | wavelet-HHH_gldm_DependenceVariance |
| 619 | wavelet-HHH_gldm_GrayLevelNonUniformity |
| 620 | wavelet-HHH_gldm_GrayLevelVariance |
| 621 | wavelet-HHH_gldm_HighGrayLevelEmphasis |
| 622 | wavelet-HHH_gldm_LargeDependenceEmphasis |
| 623 | wavelet-HHH_gldm_LargeDependenceHighGrayLevelEmphasis |
| 624 | wavelet-HHH_gldm_LargeDependenceLowGrayLevelEmphasis |
| 625 | wavelet-HHH_gldm_LowGrayLevelEmphasis |
| 626 | wavelet-HHH_gldm_SmallDependenceEmphasis |
| 627 | wavelet-HHH_gldm_SmallDependenceHighGrayLevelEmphasis |
| 628 | wavelet-HHH_gldm_SmallDependenceLowGrayLevelEmphasis |
| 629 | wavelet-HHH_glrlm_GrayLevelNonUniformity |
| 630 | wavelet-HHH_glrlm_GrayLevelNonUniformityNormalized |
| 631 | wavelet-HHH_glrlm_GrayLevelVariance |
| 632 | wavelet-HHH_glrlm_HighGrayLevelRunEmphasis |
| 633 | wavelet-HHH_glrlm_LongRunEmphasis |
| 634 | wavelet-HHH_glrlm_LongRunHighGrayLevelEmphasis |
| 635 | wavelet-HHH_glrlm_LongRunLowGrayLevelEmphasis |
| 636 | wavelet-HHH_glrlm_LowGrayLevelRunEmphasis |
| 637 | wavelet-HHH_glrlm_RunEntropy |
| 638 | wavelet-HHH_glrlm_RunLengthNonUniformity |
| 639 | wavelet-HHH_glrlm_RunLengthNonUniformityNormalized |
| 640 | wavelet-HHH_glrlm_RunPercentage |
| 641 | wavelet-HHH_glrlm_RunVariance |
| 642 | wavelet-HHH_glrlm_ShortRunEmphasis |
| 643 | wavelet-HHH_glrlm_ShortRunHighGrayLevelEmphasis |
| 644 | wavelet-HHH_glrlm_ShortRunLowGrayLevelEmphasis |
| 645 | wavelet-HHH_glszm_GrayLevelNonUniformity |
| 646 | wavelet-HHH_glszm_GrayLevelNonUniformityNormalized |
| 647 | wavelet-HHH_glszm_GrayLevelVariance |
| 648 | wavelet-HHH_glszm_HighGrayLevelZoneEmphasis |
| 649 | wavelet-HHH_glszm_LargeAreaEmphasis |
| 650 | wavelet-HHH_glszm_LargeAreaHighGrayLevelEmphasis |
| 651 | wavelet-HHH_glszm_LargeAreaLowGrayLevelEmphasis |
| 652 | wavelet-HHH_glszm_LowGrayLevelZoneEmphasis |
| 653 | wavelet-HHH_glszm_SizeZoneNonUniformity |
| 654 | wavelet-HHH_glszm_SizeZoneNonUniformityNormalized |
| 655 | wavelet-HHH_glszm_SmallAreaEmphasis |
| 656 | wavelet-HHH_glszm_SmallAreaHighGrayLevelEmphasis |
| 657 | wavelet-HHH_glszm_SmallAreaLowGrayLevelEmphasis |
| 658 | wavelet-HHH_glszm_ZoneEntropy |
| 659 | wavelet-HHH_glszm_ZonePercentage |
| 660 | wavelet-HHH_glszm_ZoneVariance |
| 661 | wavelet-HHH_ngtdm_Busyness |
| 662 | wavelet-HHH_ngtdm_Coarseness |
| 663 | wavelet-HHH_ngtdm_Complexity |
| 664 | wavelet-HHH_ngtdm_Contrast |
| 665 | wavelet-HHH_ngtdm_Strength |
| 666 | wavelet-HHL_firstorder_10Percentile |
| 667 | wavelet-HHL_firstorder_90Percentile |
| 668 | wavelet-HHL_firstorder_Energy |
| 669 | wavelet-HHL_firstorder_Entropy |
| 670 | wavelet-HHL_firstorder_InterquartileRange |
| 671 | wavelet-HHL_firstorder_Kurtosis |
| 672 | wavelet-HHL_firstorder_Maximum |
| 673 | wavelet-HHL_firstorder_Mean |
| 674 | wavelet-HHL_firstorder_MeanAbsoluteDeviation |
| 675 | wavelet-HHL_firstorder_Median |
| 676 | wavelet-HHL_firstorder_Minimum |
| 677 | wavelet-HHL_firstorder_Range |
| 678 | wavelet-HHL_firstorder_RobustMeanAbsoluteDeviation |
| 679 | wavelet-HHL_firstorder_RootMeanSquared |
| 680 | wavelet-HHL_firstorder_Skewness |
| 681 | wavelet-HHL_firstorder_TotalEnergy |
| 682 | wavelet-HHL_firstorder_Uniformity |
| 683 | wavelet-HHL_firstorder_Variance |
| 684 | wavelet-HHL_glcm_Autocorrelation |
| 685 | wavelet-HHL_glcm_ClusterProminence |
| 686 | wavelet-HHL_glcm_ClusterShade |
| 687 | wavelet-HHL_glcm_ClusterTendency |
| 688 | wavelet-HHL_glcm_Contrast |
| 689 | wavelet-HHL_glcm_Correlation |
| 690 | wavelet-HHL_glcm_DifferenceAverage |
| 691 | wavelet-HHL_glcm_DifferenceEntropy |
| 692 | wavelet-HHL_glcm_DifferenceVariance |
| 693 | wavelet-HHL_glcm_Id |
| 694 | wavelet-HHL_glcm_Idm |
| 695 | wavelet-HHL_glcm_Idmn |
| 696 | wavelet-HHL_glcm_Idn |
| 697 | wavelet-HHL_glcm_Imc1 |
| 698 | wavelet-HHL_glcm_Imc2 |
| 699 | wavelet-HHL_glcm_InverseVariance |
| 700 | wavelet-HHL_glcm_JointAverage |
| 701 | wavelet-HHL_glcm_JointEnergy |
| 702 | wavelet-HHL_glcm_JointEntropy |
| 703 | wavelet-HHL_glcm_MaximumProbability |
| 704 | wavelet-HHL_glcm_MCC |
| 705 | wavelet-HHL_glcm_SumAverage |
| 706 | wavelet-HHL_glcm_SumEntropy |
| 707 | wavelet-HHL_glcm_SumSquares |
| 708 | wavelet-HHL_gldm_DependenceEntropy |
| 709 | wavelet-HHL_gldm_DependenceNonUniformity |
| 710 | wavelet-HHL_gldm_DependenceNonUniformityNormalized |
| 711 | wavelet-HHL_gldm_DependenceVariance |
| 712 | wavelet-HHL_gldm_GrayLevelNonUniformity |
| 713 | wavelet-HHL_gldm_GrayLevelVariance |
| 714 | wavelet-HHL_gldm_HighGrayLevelEmphasis |
| 715 | wavelet-HHL_gldm_LargeDependenceEmphasis |
| 716 | wavelet-HHL_gldm_LargeDependenceHighGrayLevelEmphasis |
| 717 | wavelet-HHL_gldm_LargeDependenceLowGrayLevelEmphasis |
| 718 | wavelet-HHL_gldm_LowGrayLevelEmphasis |
| 719 | wavelet-HHL_gldm_SmallDependenceEmphasis |
| 720 | wavelet-HHL_gldm_SmallDependenceHighGrayLevelEmphasis |
| 721 | wavelet-HHL_gldm_SmallDependenceLowGrayLevelEmphasis |
| 722 | wavelet-HHL_glrlm_GrayLevelNonUniformity |
| 723 | wavelet-HHL_glrlm_GrayLevelNonUniformityNormalized |
| 724 | wavelet-HHL_glrlm_GrayLevelVariance |
| 725 | wavelet-HHL_glrlm_HighGrayLevelRunEmphasis |
| 726 | wavelet-HHL_glrlm_LongRunEmphasis |
| 727 | wavelet-HHL_glrlm_LongRunHighGrayLevelEmphasis |
| 728 | wavelet-HHL_glrlm_LongRunLowGrayLevelEmphasis |
| 729 | wavelet-HHL_glrlm_LowGrayLevelRunEmphasis |
| 730 | wavelet-HHL_glrlm_RunEntropy |
| 731 | wavelet-HHL_glrlm_RunLengthNonUniformity |
| 732 | wavelet-HHL_glrlm_RunLengthNonUniformityNormalized |
| 733 | wavelet-HHL_glrlm_RunPercentage |
| 734 | wavelet-HHL_glrlm_RunVariance |
| 735 | wavelet-HHL_glrlm_ShortRunEmphasis |
| 736 | wavelet-HHL_glrlm_ShortRunHighGrayLevelEmphasis |
| 737 | wavelet-HHL_glrlm_ShortRunLowGrayLevelEmphasis |
| 738 | wavelet-HHL_glszm_GrayLevelNonUniformity |
| 739 | wavelet-HHL_glszm_GrayLevelNonUniformityNormalized |
| 740 | wavelet-HHL_glszm_GrayLevelVariance |
| 741 | wavelet-HHL_glszm_HighGrayLevelZoneEmphasis |
| 742 | wavelet-HHL_glszm_LargeAreaEmphasis |
| 743 | wavelet-HHL_glszm_LargeAreaHighGrayLevelEmphasis |
| 744 | wavelet-HHL_glszm_LargeAreaLowGrayLevelEmphasis |
| 745 | wavelet-HHL_glszm_LowGrayLevelZoneEmphasis |
| 746 | wavelet-HHL_glszm_SizeZoneNonUniformity |
| 747 | wavelet-HHL_glszm_SizeZoneNonUniformityNormalized |
| 748 | wavelet-HHL_glszm_SmallAreaEmphasis |
| 749 | wavelet-HHL_glszm_SmallAreaHighGrayLevelEmphasis |
| 750 | wavelet-HHL_glszm_SmallAreaLowGrayLevelEmphasis |
| 751 | wavelet-HHL_glszm_ZoneEntropy |
| 752 | wavelet-HHL_glszm_ZonePercentage |
| 753 | wavelet-HHL_glszm_ZoneVariance |
| 754 | wavelet-HHL_ngtdm_Busyness |
| 755 | wavelet-HHL_ngtdm_Coarseness |
| 756 | wavelet-HHL_ngtdm_Complexity |
| 757 | wavelet-HHL_ngtdm_Contrast |
| 758 | wavelet-HHL_ngtdm_Strength |
| 759 | wavelet-HLH_firstorder_10Percentile |
| 760 | wavelet-HLH_firstorder_90Percentile |
| 761 | wavelet-HLH_firstorder_Energy |
| 762 | wavelet-HLH_firstorder_Entropy |
| 763 | wavelet-HLH_firstorder_InterquartileRange |
| 764 | wavelet-HLH_firstorder_Kurtosis |
| 765 | wavelet-HLH_firstorder_Maximum |
| 766 | wavelet-HLH_firstorder_Mean |
| 767 | wavelet-HLH_firstorder_MeanAbsoluteDeviation |
| 768 | wavelet-HLH_firstorder_Median |
| 769 | wavelet-HLH_firstorder_Minimum |
| 770 | wavelet-HLH_firstorder_Range |
| 771 | wavelet-HLH_firstorder_RobustMeanAbsoluteDeviation |
| 772 | wavelet-HLH_firstorder_RootMeanSquared |
| 773 | wavelet-HLH_firstorder_Skewness |
| 774 | wavelet-HLH_firstorder_TotalEnergy |
| 775 | wavelet-HLH_firstorder_Uniformity |
| 776 | wavelet-HLH_firstorder_Variance |
| 777 | wavelet-HLH_glcm_Autocorrelation |
| 778 | wavelet-HLH_glcm_ClusterProminence |
| 779 | wavelet-HLH_glcm_ClusterShade |
| 780 | wavelet-HLH_glcm_ClusterTendency |
| 781 | wavelet-HLH_glcm_Contrast |
| 782 | wavelet-HLH_glcm_Correlation |
| 783 | wavelet-HLH_glcm_DifferenceAverage |
| 784 | wavelet-HLH_glcm_DifferenceEntropy |
| 785 | wavelet-HLH_glcm_DifferenceVariance |
| 786 | wavelet-HLH_glcm_Id |
| 787 | wavelet-HLH_glcm_Idm |
| 788 | wavelet-HLH_glcm_Idmn |
| 789 | wavelet-HLH_glcm_Idn |
| 790 | wavelet-HLH_glcm_Imc1 |
| 791 | wavelet-HLH_glcm_Imc2 |
| 792 | wavelet-HLH_glcm_InverseVariance |
| 793 | wavelet-HLH_glcm_JointAverage |
| 794 | wavelet-HLH_glcm_JointEnergy |
| 795 | wavelet-HLH_glcm_JointEntropy |
| 796 | wavelet-HLH_glcm_MaximumProbability |
| 797 | wavelet-HLH_glcm_MCC |
| 798 | wavelet-HLH_glcm_SumAverage |
| 799 | wavelet-HLH_glcm_SumEntropy |
| 800 | wavelet-HLH_glcm_SumSquares |
| 801 | wavelet-HLH_gldm_DependenceEntropy |
| 802 | wavelet-HLH_gldm_DependenceNonUniformity |
| 803 | wavelet-HLH_gldm_DependenceNonUniformityNormalized |
| 804 | wavelet-HLH_gldm_DependenceVariance |
| 805 | wavelet-HLH_gldm_GrayLevelNonUniformity |
| 806 | wavelet-HLH_gldm_GrayLevelVariance |
| 807 | wavelet-HLH_gldm_HighGrayLevelEmphasis |
| 808 | wavelet-HLH_gldm_LargeDependenceEmphasis |
| 809 | wavelet-HLH_gldm_LargeDependenceHighGrayLevelEmphasis |
| 810 | wavelet-HLH_gldm_LargeDependenceLowGrayLevelEmphasis |
| 811 | wavelet-HLH_gldm_LowGrayLevelEmphasis |
| 812 | wavelet-HLH_gldm_SmallDependenceEmphasis |
| 813 | wavelet-HLH_gldm_SmallDependenceHighGrayLevelEmphasis |
| 814 | wavelet-HLH_gldm_SmallDependenceLowGrayLevelEmphasis |
| 815 | wavelet-HLH_glrlm_GrayLevelNonUniformity |
| 816 | wavelet-HLH_glrlm_GrayLevelNonUniformityNormalized |
| 817 | wavelet-HLH_glrlm_GrayLevelVariance |
| 818 | wavelet-HLH_glrlm_HighGrayLevelRunEmphasis |
| 819 | wavelet-HLH_glrlm_LongRunEmphasis |
| 820 | wavelet-HLH_glrlm_LongRunHighGrayLevelEmphasis |
| 821 | wavelet-HLH_glrlm_LongRunLowGrayLevelEmphasis |
| 822 | wavelet-HLH_glrlm_LowGrayLevelRunEmphasis |
| 823 | wavelet-HLH_glrlm_RunEntropy |
| 824 | wavelet-HLH_glrlm_RunLengthNonUniformity |
| 825 | wavelet-HLH_glrlm_RunLengthNonUniformityNormalized |
| 826 | wavelet-HLH_glrlm_RunPercentage |
| 827 | wavelet-HLH_glrlm_RunVariance |
| 828 | wavelet-HLH_glrlm_ShortRunEmphasis |
| 829 | wavelet-HLH_glrlm_ShortRunHighGrayLevelEmphasis |
| 830 | wavelet-HLH_glrlm_ShortRunLowGrayLevelEmphasis |
| 831 | wavelet-HLH_glszm_GrayLevelNonUniformity |
| 832 | wavelet-HLH_glszm_GrayLevelNonUniformityNormalized |
| 833 | wavelet-HLH_glszm_GrayLevelVariance |
| 834 | wavelet-HLH_glszm_HighGrayLevelZoneEmphasis |
| 835 | wavelet-HLH_glszm_LargeAreaEmphasis |
| 836 | wavelet-HLH_glszm_LargeAreaHighGrayLevelEmphasis |
| 837 | wavelet-HLH_glszm_LargeAreaLowGrayLevelEmphasis |
| 838 | wavelet-HLH_glszm_LowGrayLevelZoneEmphasis |
| 839 | wavelet-HLH_glszm_SizeZoneNonUniformity |
| 840 | wavelet-HLH_glszm_SizeZoneNonUniformityNormalized |
| 841 | wavelet-HLH_glszm_SmallAreaEmphasis |
| 842 | wavelet-HLH_glszm_SmallAreaHighGrayLevelEmphasis |
| 843 | wavelet-HLH_glszm_SmallAreaLowGrayLevelEmphasis |
| 844 | wavelet-HLH_glszm_ZoneEntropy |
| 845 | wavelet-HLH_glszm_ZonePercentage |
| 846 | wavelet-HLH_glszm_ZoneVariance |
| 847 | wavelet-HLH_ngtdm_Busyness |
| 848 | wavelet-HLH_ngtdm_Coarseness |
| 849 | wavelet-HLH_ngtdm_Complexity |
| 850 | wavelet-HLH_ngtdm_Contrast |
| 851 | wavelet-HLH_ngtdm_Strength |
| 852 | wavelet-HLL_firstorder_10Percentile |
| 853 | wavelet-HLL_firstorder_90Percentile |
| 854 | wavelet-HLL_firstorder_Energy |
| 855 | wavelet-HLL_firstorder_Entropy |
| 856 | wavelet-HLL_firstorder_InterquartileRange |
| 857 | wavelet-HLL_firstorder_Kurtosis |
| 858 | wavelet-HLL_firstorder_Maximum |
| 859 | wavelet-HLL_firstorder_Mean |
| 860 | wavelet-HLL_firstorder_MeanAbsoluteDeviation |
| 861 | wavelet-HLL_firstorder_Median |
| 862 | wavelet-HLL_firstorder_Minimum |
| 863 | wavelet-HLL_firstorder_Range |
| 864 | wavelet-HLL_firstorder_RobustMeanAbsoluteDeviation |
| 865 | wavelet-HLL_firstorder_RootMeanSquared |
| 866 | wavelet-HLL_firstorder_Skewness |
| 867 | wavelet-HLL_firstorder_TotalEnergy |
| 868 | wavelet-HLL_firstorder_Uniformity |
| 869 | wavelet-HLL_firstorder_Variance |
| 870 | wavelet-HLL_glcm_Autocorrelation |
| 871 | wavelet-HLL_glcm_ClusterProminence |
| 872 | wavelet-HLL_glcm_ClusterShade |
| 873 | wavelet-HLL_glcm_ClusterTendency |
| 874 | wavelet-HLL_glcm_Contrast |
| 875 | wavelet-HLL_glcm_Correlation |
| 876 | wavelet-HLL_glcm_DifferenceAverage |
| 877 | wavelet-HLL_glcm_DifferenceEntropy |
| 878 | wavelet-HLL_glcm_DifferenceVariance |
| 879 | wavelet-HLL_glcm_Id |
| 880 | wavelet-HLL_glcm_Idm |
| 881 | wavelet-HLL_glcm_Idmn |
| 882 | wavelet-HLL_glcm_Idn |
| 883 | wavelet-HLL_glcm_Imc1 |
| 884 | wavelet-HLL_glcm_Imc2 |
| 885 | wavelet-HLL_glcm_InverseVariance |
| 886 | wavelet-HLL_glcm_JointAverage |
| 887 | wavelet-HLL_glcm_JointEnergy |
| 888 | wavelet-HLL_glcm_JointEntropy |
| 889 | wavelet-HLL_glcm_MaximumProbability |
| 890 | wavelet-HLL_glcm_MCC |
| 891 | wavelet-HLL_glcm_SumAverage |
| 892 | wavelet-HLL_glcm_SumEntropy |
| 893 | wavelet-HLL_glcm_SumSquares |
| 894 | wavelet-HLL_gldm_DependenceEntropy |
| 895 | wavelet-HLL_gldm_DependenceNonUniformity |
| 896 | wavelet-HLL_gldm_DependenceNonUniformityNormalized |
| 897 | wavelet-HLL_gldm_DependenceVariance |
| 898 | wavelet-HLL_gldm_GrayLevelNonUniformity |
| 899 | wavelet-HLL_gldm_GrayLevelVariance |
| 900 | wavelet-HLL_gldm_HighGrayLevelEmphasis |
| 901 | wavelet-HLL_gldm_LargeDependenceEmphasis |
| 902 | wavelet-HLL_gldm_LargeDependenceHighGrayLevelEmphasis |
| 903 | wavelet-HLL_gldm_LargeDependenceLowGrayLevelEmphasis |
| 904 | wavelet-HLL_gldm_LowGrayLevelEmphasis |
| 905 | wavelet-HLL_gldm_SmallDependenceEmphasis |
| 906 | wavelet-HLL_gldm_SmallDependenceHighGrayLevelEmphasis |
| 907 | wavelet-HLL_gldm_SmallDependenceLowGrayLevelEmphasis |
| 908 | wavelet-HLL_glrlm_GrayLevelNonUniformity |
| 909 | wavelet-HLL_glrlm_GrayLevelNonUniformityNormalized |
| 910 | wavelet-HLL_glrlm_GrayLevelVariance |
| 911 | wavelet-HLL_glrlm_HighGrayLevelRunEmphasis |
| 912 | wavelet-HLL_glrlm_LongRunEmphasis |
| 913 | wavelet-HLL_glrlm_LongRunHighGrayLevelEmphasis |
| 914 | wavelet-HLL_glrlm_LongRunLowGrayLevelEmphasis |
| 915 | wavelet-HLL_glrlm_LowGrayLevelRunEmphasis |
| 916 | wavelet-HLL_glrlm_RunEntropy |
| 917 | wavelet-HLL_glrlm_RunLengthNonUniformity |
| 918 | wavelet-HLL_glrlm_RunLengthNonUniformityNormalized |
| 919 | wavelet-HLL_glrlm_RunPercentage |
| 920 | wavelet-HLL_glrlm_RunVariance |
| 921 | wavelet-HLL_glrlm_ShortRunEmphasis |
| 922 | wavelet-HLL_glrlm_ShortRunHighGrayLevelEmphasis |
| 923 | wavelet-HLL_glrlm_ShortRunLowGrayLevelEmphasis |
| 924 | wavelet-HLL_glszm_GrayLevelNonUniformity |
| 925 | wavelet-HLL_glszm_GrayLevelNonUniformityNormalized |
| 926 | wavelet-HLL_glszm_GrayLevelVariance |
| 927 | wavelet-HLL_glszm_HighGrayLevelZoneEmphasis |
| 928 | wavelet-HLL_glszm_LargeAreaEmphasis |
| 929 | wavelet-HLL_glszm_LargeAreaHighGrayLevelEmphasis |
| 930 | wavelet-HLL_glszm_LargeAreaLowGrayLevelEmphasis |
| 931 | wavelet-HLL_glszm_LowGrayLevelZoneEmphasis |
| 932 | wavelet-HLL_glszm_SizeZoneNonUniformity |
| 933 | wavelet-HLL_glszm_SizeZoneNonUniformityNormalized |
| 934 | wavelet-HLL_glszm_SmallAreaEmphasis |
| 935 | wavelet-HLL_glszm_SmallAreaHighGrayLevelEmphasis |
| 936 | wavelet-HLL_glszm_SmallAreaLowGrayLevelEmphasis |
| 937 | wavelet-HLL_glszm_ZoneEntropy |
| 938 | wavelet-HLL_glszm_ZonePercentage |
| 939 | wavelet-HLL_glszm_ZoneVariance |
| 940 | wavelet-HLL_ngtdm_Busyness |
| 941 | wavelet-HLL_ngtdm_Coarseness |
| 942 | wavelet-HLL_ngtdm_Complexity |
| 943 | wavelet-HLL_ngtdm_Contrast |
| 944 | wavelet-HLL_ngtdm_Strength |
| 945 | wavelet-LHH_firstorder_10Percentile |
| 946 | wavelet-LHH_firstorder_90Percentile |
| 947 | wavelet-LHH_firstorder_Energy |
| 948 | wavelet-LHH_firstorder_Entropy |
| 949 | wavelet-LHH_firstorder_InterquartileRange |
| 950 | wavelet-LHH_firstorder_Kurtosis |
| 951 | wavelet-LHH_firstorder_Maximum |
| 952 | wavelet-LHH_firstorder_Mean |
| 953 | wavelet-LHH_firstorder_MeanAbsoluteDeviation |
| 954 | wavelet-LHH_firstorder_Median |
| 955 | wavelet-LHH_firstorder_Minimum |
| 956 | wavelet-LHH_firstorder_Range |
| 957 | wavelet-LHH_firstorder_RobustMeanAbsoluteDeviation |
| 958 | wavelet-LHH_firstorder_RootMeanSquared |
| 959 | wavelet-LHH_firstorder_Skewness |
| 960 | wavelet-LHH_firstorder_TotalEnergy |
| 961 | wavelet-LHH_firstorder_Uniformity |
| 962 | wavelet-LHH_firstorder_Variance |
| 963 | wavelet-LHH_glcm_Autocorrelation |
| 964 | wavelet-LHH_glcm_ClusterProminence |
| 965 | wavelet-LHH_glcm_ClusterShade |
| 966 | wavelet-LHH_glcm_ClusterTendency |
| 967 | wavelet-LHH_glcm_Contrast |
| 968 | wavelet-LHH_glcm_Correlation |
| 969 | wavelet-LHH_glcm_DifferenceAverage |
| 970 | wavelet-LHH_glcm_DifferenceEntropy |
| 971 | wavelet-LHH_glcm_DifferenceVariance |
| 972 | wavelet-LHH_glcm_Id |
| 973 | wavelet-LHH_glcm_Idm |
| 974 | wavelet-LHH_glcm_Idmn |
| 975 | wavelet-LHH_glcm_Idn |
| 976 | wavelet-LHH_glcm_Imc1 |
| 977 | wavelet-LHH_glcm_Imc2 |
| 978 | wavelet-LHH_glcm_InverseVariance |
| 979 | wavelet-LHH_glcm_JointAverage |
| 980 | wavelet-LHH_glcm_JointEnergy |
| 981 | wavelet-LHH_glcm_JointEntropy |
| 982 | wavelet-LHH_glcm_MaximumProbability |
| 983 | wavelet-LHH_glcm_MCC |
| 984 | wavelet-LHH_glcm_SumAverage |
| 985 | wavelet-LHH_glcm_SumEntropy |
| 986 | wavelet-LHH_glcm_SumSquares |
| 987 | wavelet-LHH_gldm_DependenceEntropy |
| 988 | wavelet-LHH_gldm_DependenceNonUniformity |
| 989 | wavelet-LHH_gldm_DependenceNonUniformityNormalized |
| 990 | wavelet-LHH_gldm_DependenceVariance |
| 991 | wavelet-LHH_gldm_GrayLevelNonUniformity |
| 992 | wavelet-LHH_gldm_GrayLevelVariance |
| 993 | wavelet-LHH_gldm_HighGrayLevelEmphasis |
| 994 | wavelet-LHH_gldm_LargeDependenceEmphasis |
| 995 | wavelet-LHH_gldm_LargeDependenceHighGrayLevelEmphasis |
| 996 | wavelet-LHH_gldm_LargeDependenceLowGrayLevelEmphasis |
| 997 | wavelet-LHH_gldm_LowGrayLevelEmphasis |
| 998 | wavelet-LHH_gldm_SmallDependenceEmphasis |
| 999 | wavelet-LHH_gldm_SmallDependenceHighGrayLevelEmphasis |
| 1000 | wavelet-LHH_gldm_SmallDependenceLowGrayLevelEmphasis |
| 1001 | wavelet-LHH_glrlm_GrayLevelNonUniformity |
| 1002 | wavelet-LHH_glrlm_GrayLevelNonUniformityNormalized |
| 1003 | wavelet-LHH_glrlm_GrayLevelVariance |
| 1004 | wavelet-LHH_glrlm_HighGrayLevelRunEmphasis |
| 1005 | wavelet-LHH_glrlm_LongRunEmphasis |
| 1006 | wavelet-LHH_glrlm_LongRunHighGrayLevelEmphasis |
| 1007 | wavelet-LHH_glrlm_LongRunLowGrayLevelEmphasis |
| 1008 | wavelet-LHH_glrlm_LowGrayLevelRunEmphasis |
| 1009 | wavelet-LHH_glrlm_RunEntropy |
| 1010 | wavelet-LHH_glrlm_RunLengthNonUniformity |
| 1011 | wavelet-LHH_glrlm_RunLengthNonUniformityNormalized |
| 1012 | wavelet-LHH_glrlm_RunPercentage |
| 1013 | wavelet-LHH_glrlm_RunVariance |
| 1014 | wavelet-LHH_glrlm_ShortRunEmphasis |
| 1015 | wavelet-LHH_glrlm_ShortRunHighGrayLevelEmphasis |
| 1016 | wavelet-LHH_glrlm_ShortRunLowGrayLevelEmphasis |
| 1017 | wavelet-LHH_glszm_GrayLevelNonUniformity |
| 1018 | wavelet-LHH_glszm_GrayLevelNonUniformityNormalized |
| 1019 | wavelet-LHH_glszm_GrayLevelVariance |
| 1020 | wavelet-LHH_glszm_HighGrayLevelZoneEmphasis |
| 1021 | wavelet-LHH_glszm_LargeAreaEmphasis |
| 1022 | wavelet-LHH_glszm_LargeAreaHighGrayLevelEmphasis |
| 1023 | wavelet-LHH_glszm_LargeAreaLowGrayLevelEmphasis |
| 1024 | wavelet-LHH_glszm_LowGrayLevelZoneEmphasis |
| 1025 | wavelet-LHH_glszm_SizeZoneNonUniformity |
| 1026 | wavelet-LHH_glszm_SizeZoneNonUniformityNormalized |
| 1027 | wavelet-LHH_glszm_SmallAreaEmphasis |
| 1028 | wavelet-LHH_glszm_SmallAreaHighGrayLevelEmphasis |
| 1029 | wavelet-LHH_glszm_SmallAreaLowGrayLevelEmphasis |
| 1030 | wavelet-LHH_glszm_ZoneEntropy |
| 1031 | wavelet-LHH_glszm_ZonePercentage |
| 1032 | wavelet-LHH_glszm_ZoneVariance |
| 1033 | wavelet-LHH_ngtdm_Busyness |
| 1034 | wavelet-LHH_ngtdm_Coarseness |
| 1035 | wavelet-LHH_ngtdm_Complexity |
| 1036 | wavelet-LHH_ngtdm_Contrast |
| 1037 | wavelet-LHH_ngtdm_Strength |
| 1038 | wavelet-LHL_firstorder_10Percentile |
| 1039 | wavelet-LHL_firstorder_90Percentile |
| 1040 | wavelet-LHL_firstorder_Energy |
| 1041 | wavelet-LHL_firstorder_Entropy |
| 1042 | wavelet-LHL_firstorder_InterquartileRange |
| 1043 | wavelet-LHL_firstorder_Kurtosis |
| 1044 | wavelet-LHL_firstorder_Maximum |
| 1045 | wavelet-LHL_firstorder_Mean |
| 1046 | wavelet-LHL_firstorder_MeanAbsoluteDeviation |
| 1047 | wavelet-LHL_firstorder_Median |
| 1048 | wavelet-LHL_firstorder_Minimum |
| 1049 | wavelet-LHL_firstorder_Range |
| 1050 | wavelet-LHL_firstorder_RobustMeanAbsoluteDeviation |
| 1051 | wavelet-LHL_firstorder_RootMeanSquared |
| 1052 | wavelet-LHL_firstorder_Skewness |
| 1053 | wavelet-LHL_firstorder_TotalEnergy |
| 1054 | wavelet-LHL_firstorder_Uniformity |
| 1055 | wavelet-LHL_firstorder_Variance |
| 1056 | wavelet-LHL_glcm_Autocorrelation |
| 1057 | wavelet-LHL_glcm_ClusterProminence |
| 1058 | wavelet-LHL_glcm_ClusterShade |
| 1059 | wavelet-LHL_glcm_ClusterTendency |
| 1060 | wavelet-LHL_glcm_Contrast |
| 1061 | wavelet-LHL_glcm_Correlation |
| 1062 | wavelet-LHL_glcm_DifferenceAverage |
| 1063 | wavelet-LHL_glcm_DifferenceEntropy |
| 1064 | wavelet-LHL_glcm_DifferenceVariance |
| 1065 | wavelet-LHL_glcm_Id |
| 1066 | wavelet-LHL_glcm_Idm |
| 1067 | wavelet-LHL_glcm_Idmn |
| 1068 | wavelet-LHL_glcm_Idn |
| 1069 | wavelet-LHL_glcm_Imc1 |
| 1070 | wavelet-LHL_glcm_Imc2 |
| 1071 | wavelet-LHL_glcm_InverseVariance |
| 1072 | wavelet-LHL_glcm_JointAverage |
| 1073 | wavelet-LHL_glcm_JointEnergy |
| 1074 | wavelet-LHL_glcm_JointEntropy |
| 1075 | wavelet-LHL_glcm_MaximumProbability |
| 1076 | wavelet-LHL_glcm_MCC |
| 1077 | wavelet-LHL_glcm_SumAverage |
| 1078 | wavelet-LHL_glcm_SumEntropy |
| 1079 | wavelet-LHL_glcm_SumSquares |
| 1080 | wavelet-LHL_gldm_DependenceEntropy |
| 1081 | wavelet-LHL_gldm_DependenceNonUniformity |
| 1082 | wavelet-LHL_gldm_DependenceNonUniformityNormalized |
| 1083 | wavelet-LHL_gldm_DependenceVariance |
| 1084 | wavelet-LHL_gldm_GrayLevelNonUniformity |
| 1085 | wavelet-LHL_gldm_GrayLevelVariance |
| 1086 | wavelet-LHL_gldm_HighGrayLevelEmphasis |
| 1087 | wavelet-LHL_gldm_LargeDependenceEmphasis |
| 1088 | wavelet-LHL_gldm_LargeDependenceHighGrayLevelEmphasis |
| 1089 | wavelet-LHL_gldm_LargeDependenceLowGrayLevelEmphasis |
| 1090 | wavelet-LHL_gldm_LowGrayLevelEmphasis |
| 1091 | wavelet-LHL_gldm_SmallDependenceEmphasis |
| 1092 | wavelet-LHL_gldm_SmallDependenceHighGrayLevelEmphasis |
| 1093 | wavelet-LHL_gldm_SmallDependenceLowGrayLevelEmphasis |
| 1094 | wavelet-LHL_glrlm_GrayLevelNonUniformity |
| 1095 | wavelet-LHL_glrlm_GrayLevelNonUniformityNormalized |
| 1096 | wavelet-LHL_glrlm_GrayLevelVariance |
| 1097 | wavelet-LHL_glrlm_HighGrayLevelRunEmphasis |
| 1098 | wavelet-LHL_glrlm_LongRunEmphasis |
| 1099 | wavelet-LHL_glrlm_LongRunHighGrayLevelEmphasis |
| 1100 | wavelet-LHL_glrlm_LongRunLowGrayLevelEmphasis |
| 1101 | wavelet-LHL_glrlm_LowGrayLevelRunEmphasis |
| 1102 | wavelet-LHL_glrlm_RunEntropy |
| 1103 | wavelet-LHL_glrlm_RunLengthNonUniformity |
| 1104 | wavelet-LHL_glrlm_RunLengthNonUniformityNormalized |
| 1105 | wavelet-LHL_glrlm_RunPercentage |
| 1106 | wavelet-LHL_glrlm_RunVariance |
| 1107 | wavelet-LHL_glrlm_ShortRunEmphasis |
| 1108 | wavelet-LHL_glrlm_ShortRunHighGrayLevelEmphasis |
| 1109 | wavelet-LHL_glrlm_ShortRunLowGrayLevelEmphasis |
| 1110 | wavelet-LHL_glszm_GrayLevelNonUniformity |
| 1111 | wavelet-LHL_glszm_GrayLevelNonUniformityNormalized |
| 1112 | wavelet-LHL_glszm_GrayLevelVariance |
| 1113 | wavelet-LHL_glszm_HighGrayLevelZoneEmphasis |
| 1114 | wavelet-LHL_glszm_LargeAreaEmphasis |
| 1115 | wavelet-LHL_glszm_LargeAreaHighGrayLevelEmphasis |
| 1116 | wavelet-LHL_glszm_LargeAreaLowGrayLevelEmphasis |
| 1117 | wavelet-LHL_glszm_LowGrayLevelZoneEmphasis |
| 1118 | wavelet-LHL_glszm_SizeZoneNonUniformity |
| 1119 | wavelet-LHL_glszm_SizeZoneNonUniformityNormalized |
| 1120 | wavelet-LHL_glszm_SmallAreaEmphasis |
| 1121 | wavelet-LHL_glszm_SmallAreaHighGrayLevelEmphasis |
| 1122 | wavelet-LHL_glszm_SmallAreaLowGrayLevelEmphasis |
| 1123 | wavelet-LHL_glszm_ZoneEntropy |
| 1124 | wavelet-LHL_glszm_ZonePercentage |
| 1125 | wavelet-LHL_glszm_ZoneVariance |
| 1126 | wavelet-LHL_ngtdm_Busyness |
| 1127 | wavelet-LHL_ngtdm_Coarseness |
| 1128 | wavelet-LHL_ngtdm_Complexity |
| 1129 | wavelet-LHL_ngtdm_Contrast |
| 1130 | wavelet-LHL_ngtdm_Strength |
| 1131 | wavelet-LLH_firstorder_10Percentile |
| 1132 | wavelet-LLH_firstorder_90Percentile |
| 1133 | wavelet-LLH_firstorder_Energy |
| 1134 | wavelet-LLH_firstorder_Entropy |
| 1135 | wavelet-LLH_firstorder_InterquartileRange |
| 1136 | wavelet-LLH_firstorder_Kurtosis |
| 1137 | wavelet-LLH_firstorder_Maximum |
| 1138 | wavelet-LLH_firstorder_Mean |
| 1139 | wavelet-LLH_firstorder_MeanAbsoluteDeviation |
| 1140 | wavelet-LLH_firstorder_Median |
| 1141 | wavelet-LLH_firstorder_Minimum |
| 1142 | wavelet-LLH_firstorder_Range |
| 1143 | wavelet-LLH_firstorder_RobustMeanAbsoluteDeviation |
| 1144 | wavelet-LLH_firstorder_RootMeanSquared |
| 1145 | wavelet-LLH_firstorder_Skewness |
| 1146 | wavelet-LLH_firstorder_TotalEnergy |
| 1147 | wavelet-LLH_firstorder_Uniformity |
| 1148 | wavelet-LLH_firstorder_Variance |
| 1149 | wavelet-LLH_glcm_Autocorrelation |
| 1150 | wavelet-LLH_glcm_ClusterProminence |
| 1151 | wavelet-LLH_glcm_ClusterShade |
| 1152 | wavelet-LLH_glcm_ClusterTendency |
| 1153 | wavelet-LLH_glcm_Contrast |
| 1154 | wavelet-LLH_glcm_Correlation |
| 1155 | wavelet-LLH_glcm_DifferenceAverage |
| 1156 | wavelet-LLH_glcm_DifferenceEntropy |
| 1157 | wavelet-LLH_glcm_DifferenceVariance |
| 1158 | wavelet-LLH_glcm_Id |
| 1159 | wavelet-LLH_glcm_Idm |
| 1160 | wavelet-LLH_glcm_Idmn |
| 1161 | wavelet-LLH_glcm_Idn |
| 1162 | wavelet-LLH_glcm_Imc1 |
| 1163 | wavelet-LLH_glcm_Imc2 |
| 1164 | wavelet-LLH_glcm_InverseVariance |
| 1165 | wavelet-LLH_glcm_JointAverage |
| 1166 | wavelet-LLH_glcm_JointEnergy |
| 1167 | wavelet-LLH_glcm_JointEntropy |
| 1168 | wavelet-LLH_glcm_MaximumProbability |
| 1169 | wavelet-LLH_glcm_MCC |
| 1170 | wavelet-LLH_glcm_SumAverage |
| 1171 | wavelet-LLH_glcm_SumEntropy |
| 1172 | wavelet-LLH_glcm_SumSquares |
| 1173 | wavelet-LLH_gldm_DependenceEntropy |
| 1174 | wavelet-LLH_gldm_DependenceNonUniformity |
| 1175 | wavelet-LLH_gldm_DependenceNonUniformityNormalized |
| 1176 | wavelet-LLH_gldm_DependenceVariance |
| 1177 | wavelet-LLH_gldm_GrayLevelNonUniformity |
| 1178 | wavelet-LLH_gldm_GrayLevelVariance |
| 1179 | wavelet-LLH_gldm_HighGrayLevelEmphasis |
| 1180 | wavelet-LLH_gldm_LargeDependenceEmphasis |
| 1181 | wavelet-LLH_gldm_LargeDependenceHighGrayLevelEmphasis |
| 1182 | wavelet-LLH_gldm_LargeDependenceLowGrayLevelEmphasis |
| 1183 | wavelet-LLH_gldm_LowGrayLevelEmphasis |
| 1184 | wavelet-LLH_gldm_SmallDependenceEmphasis |
| 1185 | wavelet-LLH_gldm_SmallDependenceHighGrayLevelEmphasis |
| 1186 | wavelet-LLH_gldm_SmallDependenceLowGrayLevelEmphasis |
| 1187 | wavelet-LLH_glrlm_GrayLevelNonUniformity |
| 1188 | wavelet-LLH_glrlm_GrayLevelNonUniformityNormalized |
| 1189 | wavelet-LLH_glrlm_GrayLevelVariance |
| 1190 | wavelet-LLH_glrlm_HighGrayLevelRunEmphasis |
| 1191 | wavelet-LLH_glrlm_LongRunEmphasis |
| 1192 | wavelet-LLH_glrlm_LongRunHighGrayLevelEmphasis |
| 1193 | wavelet-LLH_glrlm_LongRunLowGrayLevelEmphasis |
| 1194 | wavelet-LLH_glrlm_LowGrayLevelRunEmphasis |
| 1195 | wavelet-LLH_glrlm_RunEntropy |
| 1196 | wavelet-LLH_glrlm_RunLengthNonUniformity |
| 1197 | wavelet-LLH_glrlm_RunLengthNonUniformityNormalized |
| 1198 | wavelet-LLH_glrlm_RunPercentage |
| 1199 | wavelet-LLH_glrlm_RunVariance |
| 1200 | wavelet-LLH_glrlm_ShortRunEmphasis |
| 1201 | wavelet-LLH_glrlm_ShortRunHighGrayLevelEmphasis |
| 1202 | wavelet-LLH_glrlm_ShortRunLowGrayLevelEmphasis |
| 1203 | wavelet-LLH_glszm_GrayLevelNonUniformity |
| 1204 | wavelet-LLH_glszm_GrayLevelNonUniformityNormalized |
| 1205 | wavelet-LLH_glszm_GrayLevelVariance |
| 1206 | wavelet-LLH_glszm_HighGrayLevelZoneEmphasis |
| 1207 | wavelet-LLH_glszm_LargeAreaEmphasis |
| 1208 | wavelet-LLH_glszm_LargeAreaHighGrayLevelEmphasis |
| 1209 | wavelet-LLH_glszm_LargeAreaLowGrayLevelEmphasis |
| 1210 | wavelet-LLH_glszm_LowGrayLevelZoneEmphasis |
| 1211 | wavelet-LLH_glszm_SizeZoneNonUniformity |
| 1212 | wavelet-LLH_glszm_SizeZoneNonUniformityNormalized |
| 1213 | wavelet-LLH_glszm_SmallAreaEmphasis |
| 1214 | wavelet-LLH_glszm_SmallAreaHighGrayLevelEmphasis |
| 1215 | wavelet-LLH_glszm_SmallAreaLowGrayLevelEmphasis |
| 1216 | wavelet-LLH_glszm_ZoneEntropy |
| 1217 | wavelet-LLH_glszm_ZonePercentage |
| 1218 | wavelet-LLH_glszm_ZoneVariance |
| 1219 | wavelet-LLH_ngtdm_Busyness |
| 1220 | wavelet-LLH_ngtdm_Coarseness |
| 1221 | wavelet-LLH_ngtdm_Complexity |
| 1222 | wavelet-LLH_ngtdm_Contrast |
| 1223 | wavelet-LLH_ngtdm_Strength |
| 1224 | wavelet-LLL_firstorder_10Percentile |
| 1225 | wavelet-LLL_firstorder_90Percentile |
| 1226 | wavelet-LLL_firstorder_Energy |
| 1227 | wavelet-LLL_firstorder_Entropy |
| 1228 | wavelet-LLL_firstorder_InterquartileRange |
| 1229 | wavelet-LLL_firstorder_Kurtosis |
| 1230 | wavelet-LLL_firstorder_Maximum |
| 1231 | wavelet-LLL_firstorder_Mean |
| 1232 | wavelet-LLL_firstorder_MeanAbsoluteDeviation |
| 1233 | wavelet-LLL_firstorder_Median |
| 1234 | wavelet-LLL_firstorder_Minimum |
| 1235 | wavelet-LLL_firstorder_Range |
| 1236 | wavelet-LLL_firstorder_RobustMeanAbsoluteDeviation |
| 1237 | wavelet-LLL_firstorder_RootMeanSquared |
| 1238 | wavelet-LLL_firstorder_Skewness |
| 1239 | wavelet-LLL_firstorder_TotalEnergy |
| 1240 | wavelet-LLL_firstorder_Uniformity |
| 1241 | wavelet-LLL_firstorder_Variance |
| 1242 | wavelet-LLL_glcm_Autocorrelation |
| 1243 | wavelet-LLL_glcm_ClusterProminence |
| 1244 | wavelet-LLL_glcm_ClusterShade |
| 1245 | wavelet-LLL_glcm_ClusterTendency |
| 1246 | wavelet-LLL_glcm_Contrast |
| 1247 | wavelet-LLL_glcm_Correlation |
| 1248 | wavelet-LLL_glcm_DifferenceAverage |
| 1249 | wavelet-LLL_glcm_DifferenceEntropy |
| 1250 | wavelet-LLL_glcm_DifferenceVariance |
| 1251 | wavelet-LLL_glcm_Id |
| 1252 | wavelet-LLL_glcm_Idm |
| 1253 | wavelet-LLL_glcm_Idmn |
| 1254 | wavelet-LLL_glcm_Idn |
| 1255 | wavelet-LLL_glcm_Imc1 |
| 1256 | wavelet-LLL_glcm_Imc2 |
| 1257 | wavelet-LLL_glcm_InverseVariance |
| 1258 | wavelet-LLL_glcm_JointAverage |
| 1259 | wavelet-LLL_glcm_JointEnergy |
| 1260 | wavelet-LLL_glcm_JointEntropy |
| 1261 | wavelet-LLL_glcm_MaximumProbability |
| 1262 | wavelet-LLL_glcm_MCC |
| 1263 | wavelet-LLL_glcm_SumAverage |
| 1264 | wavelet-LLL_glcm_SumEntropy |
| 1265 | wavelet-LLL_glcm_SumSquares |
| 1266 | wavelet-LLL_gldm_DependenceEntropy |
| 1267 | wavelet-LLL_gldm_DependenceNonUniformity |
| 1268 | wavelet-LLL_gldm_DependenceNonUniformityNormalized |
| 1269 | wavelet-LLL_gldm_DependenceVariance |
| 1270 | wavelet-LLL_gldm_GrayLevelNonUniformity |
| 1271 | wavelet-LLL_gldm_GrayLevelVariance |
| 1272 | wavelet-LLL_gldm_HighGrayLevelEmphasis |
| 1273 | wavelet-LLL_gldm_LargeDependenceEmphasis |
| 1274 | wavelet-LLL_gldm_LargeDependenceHighGrayLevelEmphasis |
| 1275 | wavelet-LLL_gldm_LargeDependenceLowGrayLevelEmphasis |
| 1276 | wavelet-LLL_gldm_LowGrayLevelEmphasis |
| 1277 | wavelet-LLL_gldm_SmallDependenceEmphasis |
| 1278 | wavelet-LLL_gldm_SmallDependenceHighGrayLevelEmphasis |
| 1279 | wavelet-LLL_gldm_SmallDependenceLowGrayLevelEmphasis |
| 1280 | wavelet-LLL_glrlm_GrayLevelNonUniformity |
| 1281 | wavelet-LLL_glrlm_GrayLevelNonUniformityNormalized |
| 1282 | wavelet-LLL_glrlm_GrayLevelVariance |
| 1283 | wavelet-LLL_glrlm_HighGrayLevelRunEmphasis |
| 1284 | wavelet-LLL_glrlm_LongRunEmphasis |
| 1285 | wavelet-LLL_glrlm_LongRunHighGrayLevelEmphasis |
| 1286 | wavelet-LLL_glrlm_LongRunLowGrayLevelEmphasis |
| 1287 | wavelet-LLL_glrlm_LowGrayLevelRunEmphasis |
| 1288 | wavelet-LLL_glrlm_RunEntropy |
| 1289 | wavelet-LLL_glrlm_RunLengthNonUniformity |
| 1290 | wavelet-LLL_glrlm_RunLengthNonUniformityNormalized |
| 1291 | wavelet-LLL_glrlm_RunPercentage |
| 1292 | wavelet-LLL_glrlm_RunVariance |
| 1293 | wavelet-LLL_glrlm_ShortRunEmphasis |
| 1294 | wavelet-LLL_glrlm_ShortRunHighGrayLevelEmphasis |
| 1295 | wavelet-LLL_glrlm_ShortRunLowGrayLevelEmphasis |
| 1296 | wavelet-LLL_glszm_GrayLevelNonUniformity |
| 1297 | wavelet-LLL_glszm_GrayLevelNonUniformityNormalized |
| 1298 | wavelet-LLL_glszm_GrayLevelVariance |
| 1299 | wavelet-LLL_glszm_HighGrayLevelZoneEmphasis |
| 1300 | wavelet-LLL_glszm_LargeAreaEmphasis |
| 1301 | wavelet-LLL_glszm_LargeAreaHighGrayLevelEmphasis |
| 1302 | wavelet-LLL_glszm_LargeAreaLowGrayLevelEmphasis |
| 1303 | wavelet-LLL_glszm_LowGrayLevelZoneEmphasis |
| 1304 | wavelet-LLL_glszm_SizeZoneNonUniformity |
| 1305 | wavelet-LLL_glszm_SizeZoneNonUniformityNormalized |
| 1306 | wavelet-LLL_glszm_SmallAreaEmphasis |
| 1307 | wavelet-LLL_glszm_SmallAreaHighGrayLevelEmphasis |
| 1308 | wavelet-LLL_glszm_SmallAreaLowGrayLevelEmphasis |
| 1309 | wavelet-LLL_glszm_ZoneEntropy |
| 1310 | wavelet-LLL_glszm_ZonePercentage |
| 1311 | wavelet-LLL_glszm_ZoneVariance |
| 1312 | wavelet-LLL_ngtdm_Busyness |
| 1313 | wavelet-LLL_ngtdm_Coarseness |
| 1314 | wavelet-LLL_ngtdm_Complexity |
| 1315 | wavelet-LLL_ngtdm_Contrast |
| 1316 | wavelet-LLL_ngtdm_Strength |
